# Intervening Effects of Total Alkaloids of *Corydalis saxicola* Bunting on Rats With Antibiotic-Induced Gut Microbiota Dysbiosis Based on 16S rRNA Gene Sequencing and Untargeted Metabolomics Analyses

**DOI:** 10.3389/fmicb.2019.01151

**Published:** 2019-05-31

**Authors:** Xi Liu, Hua Zheng, Rigang Lu, Huimin Huang, Hongjia Zhu, Chunli Yin, Yiyi Mo, Jinxia Wu, Xuwen Liu, Ming Deng, Danfeng Li, Bang Cheng, Fang Wu, Yonghong Liang, Hongwei Guo, Hui Song, Zhiheng Su

**Affiliations:** ^1^Pharmaceutical College, Guangxi Medical University, Nanning, China; ^2^Life Science Institute, Guangxi Medical University, Nanning, China; ^3^Guangxi Institute for Food and Drug Control, Nanning, China; ^4^Wuhan Institute of Physics and Mathematics, Chinese Academy of Sciences, Wuhan, China

**Keywords:** antibiotic-induced gut microbiota dysbiosis, 16S rRNA gene sequencing analysis, untargeted metabolomics, total alkaloids of *Corydalis saxicola* Bunting (TACS), ultra-performance liquid chromatography–quadrupole time-of-flight/mass spectrometry

## Abstract

Gut microbiota dysbiosis induced by antibiotics is strongly connected with health concerns. Studying the mechanisms underlying antibiotic-induced gut microbiota dysbiosis could help to identify effective drugs and prevent many serious diseases. In this study, in rats with antibiotic-induced gut microbiota dysbiosis treated with total alkaloids of *Corydalis saxicola* Bunting (TACS), urinary and fecal biochemical changes and cecum microbial diversity were investigated using 16S rRNA gene sequencing analysis and untargeted metabolomics. The microbial diversity results showed that 10 genera were disturbed by the antibiotic treatment, and two of them were obviously restored by TACS. The untargeted metabolomics analysis identified 34 potential biomarkers in urine and feces that may be the metabolites that are most related to the mechanisms underlying antibiotic-induced gut microbiota dysbiosis and the therapeutic effects of TACS treatment. The biomarkers were involved in six metabolic pathways, comprising pathways related to branched-chain amino acid (BCAA), bile acid, arginine and proline, purine, aromatic amino acid, and amino sugar and nucleotide sugar metabolism. Notably, there was a strong correlation between these metabolic pathways and two gut microbiota genera (*g__Blautia* and *g__Intestinibacter*). The correlation analysis suggested that TACS might synergistically affect four of these metabolic pathways (BCAA, bile acid, arginine and proline, and purine metabolism), thereby modulating gut microbiota dysbiosis. Furthermore, we performed a molecular docking analysis involving simulating high-precision docking and using molecular pathway maps to illuminate the way that ligands (the five main alkaloid components of TACS) act on a complex molecular network, using CYP27A1 (a key enzyme in the bile acid synthesis pathway) as the target protein. This study provides a comprehensive overview of the intervening effects of TACS on the host metabolic phenotype and gut microbiome in rats with gut microbiota dysbiosis, and it presents new insights for the discovery of effective drugs and the best therapeutic approaches.

## Introduction

Millions of microorganisms that live in human organs have commensal relationships with humans. The microorganisms that inhabit the gastrointestinal tract are called *gut microbiota*, which are a key factor in host health and confer an extended metabolic capacity on the host (Swann et al., 2011). There are many factors that influence the gut microbiota, such as diet, age, and antibiotic administration, which can lead to microbiota depletion and result in gut microbiota dysbiosis (Dethlefsen et al., 2006; Zarrinpar et al., 2018). Gut microbiota dysbiosis is strongly associated with several serious health concerns including obesity, type 2 diabetes, and non-alcoholic fatty liver disease (Cox and Blaser, 2013; Mahana et al., 2016). Studies have shown that disease- or drug-related gut microbiota dysbiosis can be regulated by natural medicinal herbs, such as Chai-Hu-Shu-Gan-San and cloudberry (Yu et al., 2017; Carrera-Quintanar et al., 2018). Thus, investigation of the protective effects of Chinese traditional medicines against gut microbiota dysbiosis will broaden the search for effective therapeutic drugs.

The traditional Chinese medicine, total alkaloids of *Corydalis saxicola* Bunting (TACS), which comprises the major active components of *Corydalis saxicola* Bunting (CS), can be used for treating liver disease. TACS has a variety of pharmacological activities such as antibacterial, antiviral, and anticancer activities (Liang et al., 2016). Our previous study partially elucidated the mechanism of TACS for treating chronic liver injury and demonstrated that TACS for liver disease synergistically regulated the abnormalities of gut microbiota metabolism (Wu et al., 2017). The mode of action of TACS when treating liver disease indicated that it simultaneously modulated both the host co-metabolism and the gut microbiota. We therefore inferred that TACS can correct gut flora disorders. However, there are few studies on the exact intervening effects of TACS against gut microbiota dysbiosis. Animal models of antibiotic-induced gut microbiota dysbiosis have often been used to mimic certain types of disease-related gut microbiota dysbiosis, which can provide a true picture of “normal” mammalian physiology. These animal models are optimal models for characterizing microbiota mammalian metabolic interactions in “normal” animals (Willing et al., 2011).

Nowadays, 16S rRNA gene sequencing combined with untargeted metabolomics is a common method, based on gut microbiota analysis and metabolism analysis, for studying the mechanisms underlying diseases or the mechanisms underlying treatments (Li et al., 2018; Zhao X. et al., 2018). The 16S rRNA gene is present in the genome of all bacteria and is highly conserved and specific. A 16S rRNA gene sequencing analysis can rapidly, accurately, and simply classify bacteria, and it can be used to characterize the cecum microbiota profile associated with different treatments. Metabolomics, the burgeoning “omics” technique connected to the fields of genomics, transcriptomics, and proteomics, can be used to reflect the global, unbiased metabolic profile of biological samples, including urine, feces, plasma, and tissue samples, providing details of the metabolic responses of living organism to external stimuli (including pathological stimuli and drug treatments) (Griffin et al., 2000; Su et al., 2011). It can provide details of changes in metabolic profiles in animals and humans, indicating pathophysiological status and disease progression, and it also reflects diagnostic and prognostic details related to the biochemical effects of toxins and drug treatments (Wang et al., 2013; Jacob et al., 2018; Probert et al., 2018). Urine and feces are preferable biospecimens for researchers as they can be collected without risk to subjects or alterations in their components, and they provide good representations of the differences in the metabolome and gut microbial diversity between health and disease states (Bouatra et al., 2013).

In this study, a rat model of antibiotic-induced gut microbiota dysbiosis was employed to directly investigate the dynamic effects of the microbiome on the urinary and fecal metabolome. A 16S rRNA gene sequencing analysis and ultra-performance liquid chromatography (UPLC)–quadrupole time-of-flight (Q-TOF)/mass spectrometry (MS)–based metabolomics were used to provide a general understanding of the intervening effects of TACS on the host co-metabolism and gut microbiota in rats with antibiotic-induced gut microbiota dysbiosis.

## Materials and Methods

### Reagents and Chemicals

Ultrapure water (18.25 MΩ) was prepared using an ultrapure water system (Chengdu Yue Chun Technology Co., Ltd., Chengdu, China). Acetonitrile, formic acid, and methanol (all high-performance liquid chromatography [HPLC] grade) were obtained from Tedia (Fairfield, OH, United States). Ammonium formate was purchased from Thermo Fisher Scientific Inc. (Shanghai, China). The broad-spectrum *β*-lactam antibiotic imipenem/cilastatin sodium (in equal quantities; trade name: Tienam) was purchased from Hangzhou MSD Pharmaceutical Co., Ltd. (Hangzhou, China). Analytical-grade chemicals were used in our experiments.

### Preparation of TACS

The CS herb was purchased from Nanning Shengyuantang Chinese Herbal Medicine Co., Ltd. (Nanning, China), and it was authenticated by Associate Professor Changming Mo at the Guangxi Academy of Agricultural Sciences. The CS herb was cleaned, dried, and cut into small pieces. Supplementary Figure 1 shows the TACS extraction protocol in our lab. After the extraction was finished, the TACS sample was stored in a desiccator.

UPLC-Q-TOF/MS was performed to characterize the chemical profile of TACS. Corresponding analytical methods and results are described in the Supplementary Materials.

### Animal Experiments

Thirty healthy male Sprague–Dawley rats (weighing 100–140 g) were obtained from the Experimental Animal Center of Guangxi Medical University (Guangxi, China; Approval No.: SCXK-20140002). The rats were raised individually in plastic cages in a room maintained at 22 ± 3°C and 55 ± 15% humidity with a 12-h light–12-h dark cycle and free access to water. The rats were randomly divided into three groups (with 10 rats in each group): a control group, an antibiotic-only group, and a TACS group. The control group was given 1 ml/100 g i.g., normal saline. The antibiotic-only group received 50 mg/kg i.g. of the antibiotic preparation imipenem/cilastatin sodium. The TACS group received the same dose of imipenem/cilastatin sodium with 50 mg/kg oral TACS. The rats were treated once a day for 21 days (Zheng et al., 2011). The body weight was recorded daily as shown in Supplementary Figure 4.

This study was approved by the Ethics Committee for Animal Experiments of Guangxi Medical University (No. 201709003).

### Sample Collection and Preparation

On day 20, urine samples were collected for 24 h from rats in metabolic cages (with one rat per cage). The urine samples were stored at -80°C. They were thawed at room temperature before analysis and centrifuged (13,000 rpm, 4°C, 10 min). Next, 500-μl supernatant was mixed with 500-μl water, and an aliquot of 5 μl was used for UPLC-Q-TOF/MS analysis.

Feces samples were also collected on day 20 and then transferred into sterile conical tubes. The feces samples were immediately frozen in liquid nitrogen and stored at -80°C. They were ground on ice and weighed. The samples were processed by adding ice-cold water at a ratio of 100 mg/500 μl, vortexing, blending, and centrifuging (13,000 rpm, 4°C, 15 min). The supernatant was collected, and the sample was further processed using the same method with ice-cold methanol. The double-extraction mixture was twirled and centrifuged (13,000 rpm, 20 min, 4°C), and an aliquot of 5 μl was used for UPLC-Q-TOF/MS analysis.

At 24 h after the last treatment administration, all the rats were sacrificed, and cecum samples were collected in clean centrifuge tubes. The cecum samples were immediately placed in dry ice and then stored at -80°C until microbiological analysis.

### 16S rRNA Gene Sequencing Analysis

The microbial DNA was extracted from the cecum samples and prepared for 16S rRNA gene sequencing analysis. The analysis was performed as follows: (i) the microbial DNA was extracted; (ii) the DNA quality was assessed by electrophoresis; (iii) the V3–V4 hyper-variable regions of the bacterial 16S rRNA gene were amplified using a Thermocycler PCR system (Gene Amp 9700, ABI, United States); (iv) PCR products were extracted, purified, and then sequenced on an Illumina MiSeq platform (Illumina, San Diego, CA, United States); and (v) raw fastq data were processed and analyzed. Detailed steps are described in the Supplementary Materials. The raw 16S rRNA sequence data have been deposited in the National Center for Biotechnology Information (NCBI) Sequence Read Archive (SRA) database under accession number PRJNA529316.

According to the references in the Greengenes (v13.5) database, PICRUSt was performed using the abundance predictions of the Kyoto Encyclopedia of Genes and Genomes (KEGG) orthologs and the KEGG pathways of the bacterial communities (Langille et al., 2013). STAMP was used to calculate and visualize the functional differences between pairs of groups (Parks et al., 2014). We used two-sided Welch’s t-tests and the Benjamini–Hochberg false discovery rate (FDR) correction for statistical analysis in the two-group functional prediction analysis. Moreover, the prediction accuracy of PICRUSt was evaluated using the Nearest Sequenced Taxon Index (NSTI), with lower values indicating higher prediction accuracy (Hu et al., 2016).

### Metabolomics Analysis

#### UPLC-Q-TOF/MS Analysis

Chromatographic analysis of urine and feces samples was performed using an ACQUITY UPLC BEH C18 column (100 × 2.1 mm, 1.7 μm) with a Waters ACQUITY UPLC system (Waters Corp., Milford, MA, United States). The temperature of the columns was set at 40°C, and the flow rate was 0.40 ml/min. For the urine samples, we used water (A) and acetonitrile (B), each containing 0.1% formic acid as the mobile phase. The gradient program for the urine samples was optimized as follows: 0–0.5 min, 2% B; 0.5–6 min, 1% B to 20% B; 6–12 min, 20% B to 100% B; 12–16 min, washing with 100% B; 16–18 min, 100% B to 2% B; 18–25 min, equilibration with 2% B. For the feces samples, we used 2 mM ammonium formate in 95% H_2_O/5% acetonitrile + 0.1% formic acid (A) and 2 mM ammonium formate in 95% acetonitrile/5% H_2_O + 0.1% formic acid (B) as the mobile phase. The gradient program for the fecal samples was optimized as follows: 0–0.5 min, 5% B; 0.5–5 min, 5% B to 40% B; 5–11 min, 40% B to 70% B; 11–12 min, 70% B to 100% B; 12–19 min, washing with 100% B; 19–20 min, 100% B to 5% B; 20–25 min equilibration with 5% B. The eluent from the column was directed to the mass spectrometer with no splitting.

Mass spectrometry with an electrospray ionization source operating in positive ion mode was performed on a XEVO G2-XS QTOF mass spectrometer (Waters Corp., Manchester, United Kingdom). The parameters were set as follows: capillary voltage, 3.0 kV; sample and extraction cone voltage, 40 and 4.0 V; desolvation gas rate and temperature, 800 L/h and 400°C; cone gas rate, 40 L/h; source temperature, 100°C; scan time, 0.15 s; and interscan delay, 0.02 s. We used leucine-enkephalin as the lock mass in all analyses ([M + H]^+^ = 556.2771) at a concentration of 0.5 μg/ml with a flow rate of 5 μl/min. Data were collected in centroid mode from m/z 50 to 1500.

#### Method Validation

Quality control (QC) samples, made by pooling the same volume (10 μl) from each urine and feces samples, were prepared for ensuring the stability of the UPLC-Q-TOF/MS analysis. To evaluate the stability, we analyzed the pooled QC samples randomly during the analytical batch process. We extracted 10 ions to evaluate the repeatability of our method using six replicates of QC sample. The relative standard deviations (R.S.D.%) of the retention time and m/z are shown in Supplementary Table 2.

#### Data Processing and Multivariate Analysis

MarkerLynx Applications Manager version 4.1 (Waters, Manchester, United Kingdom) was used to analyze the raw data. After preprocessing (including integration, normalization peak and alignment), a list of intensities of the peaks were labeled on retention time, and *m/z* data pairs were obtained from the samples in the positive data set. The SIMCA-P 14.1 software package (Umetrics, Umeå, Sweden) was used to analyze the data list by performing multivariate analysis, including principal component analysis (PCA) and orthogonal partial least squares–discriminant analysis (OPLS-DA). PCA was used to verify whether the three groups could be separated, while partial least squares (PLS)-DA was performed to investigate the different metabolic profiles. OPLS-DA was carried out to enhance the differences between the antibiotic-only and control groups and to identify the different variables contributing to the differentiation between the antibiotic-only and control groups. The score plots displayed the group clusters, and the S-plot showed the variables that contributed to the classification. In the OPLS-DA, the variables that were screened as potential biomarkers of dysbiosis, based on a variable importance on projection (VIP) value > 1 and *p* < 0.05, differentiated between the antibiotic-only and control groups. And seven-fold cross-validation was used to evaluate the predictive abilities of the constructed OPLS-DA model. The accurate masses of compounds, which were obtained from online resources such as the Human Metabolome Database (HMDB)^1^, Massbank^2^, and METLIN^3^, were used for identification. Heat maps were used to visualize the potential biomarkers, and Spearman’s correlation analyses between the potential markers and the significantly changed gut microbiota levels were performed using MetaboAnalyst. All potential biomarkers identified in this study using the Kyoto Encyclopedia of Genes and Genomes (KEGG)^4^, HMDB, and MetaboAnalyst^5^ were integrated with each other in order to map the comprehensive metabolic network.

### Molecular Docking

Using high-precision docking simulations and molecular pathway maps, we performed a molecular docking analysis to comprehensively characterize the selectivity of five ligands and to illustrate how they act on a complex molecular network. The target protein in the analysis was CYP27A1 (Protein Data Bank [PDB]-ID: 1mfx), which is a key enzyme in the bile acid synthesis pathway. The chemical structures of the five ligands, which are the main alkaloid components of TACS (berberine, palmatine, chelerythrine, jatrorrhizine, and dehydrocavidine), are shown in Supplementary Figure 5. The molecular docking analysis was carried out using the Surflex dock module of the licensed software SYBYL-X version 2.0 (Jain, 2007) as follows: (1) the .mol2 format documents of chemical structures were prepared by SYBYL-X 2.0; (2) Tripos force field was used for the energy minimization calculation to optimize the structure regarding the combination of each ligand and the target protein; (3) the protein data were downloaded from PDB^6^; (4) the protein was prepared using SYBYL-X 2.0 by removing the substructure (chain B) and water, fixing the bumps, and incorporating hydrogen; and (5) the molecular docking analysis was implemented (Liang et al., 2018; Thillainayagam et al., 2018). The molecular conformations identified from the molecular docking analysis were modified using the molecular conformation based on the crystal structure in PyMOL software (Seeliger and de Groot, 2010).

One-way analysis of variance (ANOVA) was used for evaluating the between-group significant differences in potential biomarkers using Statistical Package for Social Sciences (SPSS) program software version 16.0 (Chicago, IL, United States). *P* < 0.05 was set as the significance threshold.

## Results

### Gut Microbiota Diversity and Community Composition After Antibiotic Administration

**FIGURE 1:**
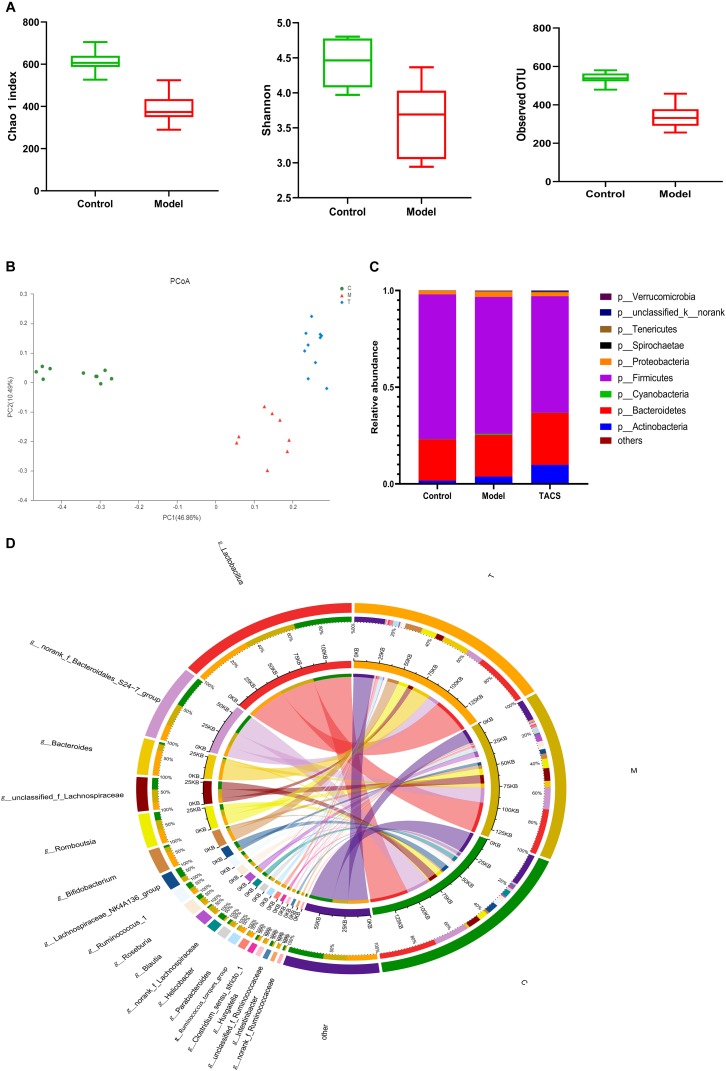
Gut microbial diversity and richness in the control and antibiotic-only groups **(A)**. Principal coordinate analysis (PCoA) **(B)** and bacterial phylum composition profiles **(C)** of gut microbiota community in the control group (C), antibiotic-only group (M), and TACS group (T). Circos plot **(D)** connecting genera detected by 16S rRNA gene sequencing with the gut microbiota profiles in the control group (C) and antibiotic-only group (M).

16S rRNA gene sequencing was performed to analyze the cecum microbiota diversity and composition. We assessed the gut microbial diversity and richness using the Chao 1 index, Shannon diversity index, and observed operational taxonomic units (OTUs). Figure 1A shows that significant changes in both diversity and richness were found after antibiotic administration. A principal coordinate analysis (PCoA) was carried out to compare the microbiota patterns in each group. The distinct separations in the scatterplot indicated the significant differences in community composition between the three groups (Figure 1B).

Around 99% of the total bacterial relative abundance was classified into nine phyla and 161 genera; *p__Firmicutes* and *p__Bacteroidetes* were the dominant phyla, followed by *p__Actinobacteria* and *p__Proteobacteria* (Figure 1C). The 19 dominant genera that had relative abundance > 1% in each group were subjected to further analysis (Figure 1D). Ten of these genera significantly changed in the antibiotic-only group (Table 1). Among these genera, *g__Blautia, g__Intestinibacter, g__Parabacteroides, g__Hungatella*, and *g__Ruminococcus_torques_group* were significantly upregulated in the antibiotic-only group compared to the control group, and the former two were obviously restored by TACS treatment. In contrast, *g__Clostridium_sensu_stricto_1, g__Ruminococcus_1, g__norank_f_Lachnospiraceae, g__unclassified_f_Ruminococcaceae*, and *g__norank_f_Ruminococcaceae* were significantly downregulated in the antibiotic-only group. Additionally, *g__Blautia* was completely restored to the control level by TACS, while there was incomplete restoration of *g__Clostridium_sensu_stricto_1*, *g__Hungatella*, and *g__Intestinibacter*. These results indicated that gut microbiota dysbiosis at the OTU level and genus level could be caused by antibiotic administration, and this kind of dysbiosis can be modulated by TACS treatment.

**Table 1:** Cecum microbiota genera that showed significant changes between the control (C), antibiotic-only (M), and TACS (T) groups detected by16S rRNA gene sequencing analysis (^∗^*p* < 0.05; ^∗∗^*p* < 0.01; ^∗∗∗^*p* < 0.001).

Genus	M vs. C	T vs. M	T vs. C
*g__Ruminococcus_torques_group*	↑	–	↑
*g__Blautia*	↑	↓^∗∗^	↓
*g__Ruminococcus_1*	↓	–	↓
*g__norank_f__Lachnospiraceae*	↓^∗∗∗^	–	↓^∗∗∗^
*g__Clostridium_sensu_stricto_1*	↓^∗∗^	↑	↓
*g__unclassified_f__Ruminococcaceae*	↓^∗∗^	–	↓^∗∗∗^
*g__Parabacteroides*	↑	↑	↑
*g__norank_f__Ruminococcaceae*	↓^∗∗∗^	–	↓^∗∗∗^
*g__Intestinibacter*	↑	↓^∗∗^	↓
*g__Hungatella*	↑	↓	↑

### Urinary Metabolic Profiles of Gut Microbiota Dysbiosis With and Without TACS Treatment

**FIGURE 2:**
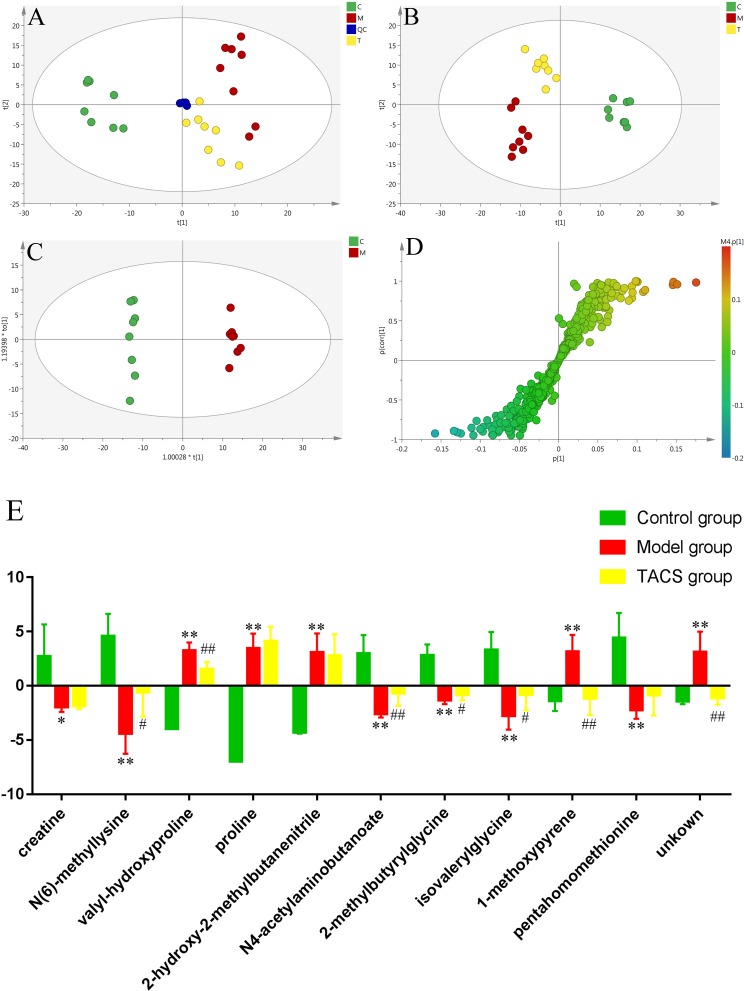
Principal component analysis (PCA) score plot of urinary samples collected from different rat treatment groups in the positive mode (**A**, *R*^2^X = 0.577, *Q*^2^(cum) = 0.365). Partial least squares–discriminant analysis (PLS-DA) score plot of urinary samples collected from different rat treatment groups in the positive mode (**B**, *R*^2^X = 0.581, *R*^2^Y = 0.983, *Q*^2^(cum) = 0.934). Orthogonal partial least squares–discriminant analysis (OPLS-DA) score plot and S-plot of the control and antibiotic-only groups in the positive mode **(C,D)**. (C), control group; (M), antibiotic-only group; (T), TACS group; and (QC), quality control group. Significant changes in urinary metabolites in different groups are shown in the histogram **(E)**. (Antibiotic-only group vs. control group: ^∗^*p <* 0.05; ^∗∗^*p <* 0.01; TACS group vs. antibiotic-only group: ^#^*p <* 0.05; ^##^*p <* 0.01).

UPLC-Q-TOF/MS in the positive ion mode was performed to investigate urine samples from the control, antibiotic-only, and TACS groups, which represented physiological status, pathological condition, and intervening effects, respectively. Typical base peak intensity (BPI) chromatograms of the three groups are shown in Supplementary Figure 6. PCA demonstrated the separation of the three groups (Figure 2A). PLS-DA, a supervised multivariable statistical method that was carried out to better visualize the discriminated groups from PCA and to evaluate the metabolic patterns of all three groups, showed that the metabolic profile of the antibiotic-only group was clearly separated from that of the control group, while the TACS group also differed from the antibiotic-only group and was similar to the control group (Figure 2B). The results indicated that antibiotic administration induced significant metabolic changes in the urine and that these changes could be modulated by TACS treatment.

### Potential Urinary Biomarkers Associated With Gut Microbiota Dysbiosis

To enhance the separation of the antibiotic-only and control groups established in the PLS-DA, OPLS-DA was employed (Figure 2C). Seven-fold cross-validation was used to evaluate the predictive abilities of the constructed OPLS-DA model. Furthermore, a 200-times permutation test was carried out between the control and antibiotic-only groups to prevent model over-fitting (Supplementary Figure 7). Significantly altered metabolites in the urine were selected using the S-plot and the VIP values. We considered the variables far from the origin in the S-plot with VIP > 1 to be the potential biomarkers associated with gut microbiota dysbiosis. As revealed in Figure 2D, 11 variables contributing to the clustering from the S-plot, based on the differences in the urine metabolic profiles between the antibiotic-only and control groups, were treated as the potential biomarkers. Their retention times, *m/z*, and VIP values are listed in Supplementary Table 3. Based on the accurate mass and the MS^E^ spectra measurements obtained *via* Q-TOF/MS and comparison of the data with literature and/or online databases, such as (HMDB), METLIN, Massbank, and KEGG, 10 identified endogenous metabolites were characterized. These metabolites were creatine, *N*(6)-methyllysine, valyl-hydroxyproline, proline, 2-hydroxy-2-methylbutanenitrile, *N*4-acetylaminobutanoate, betonicine, isovalerylglycine, 1-methoxypyrene, and pentahomomethionine.

Among these metabolites, valyl-hydroxyproline, proline, 2-hydroxy-2-methylbutanenitrile, and 1-methoxypyrene were increased significantly in the antibiotic-only rats compared to the controls, while creatine, *N*(6)-methyllysine, *N*4-acetylaminobutanoate, betonicine, isovalerylglycine, and pentahomomethionine were clearly decreased in the antibiotic-only rats. After TACS treatment, the level of *N*(6)-methyllysine, *N*4-acetylaminobutanoate, betonicine, and isovalerylglycine were upregulated compared to the antibiotic-only group, while valyl-hydroxyproline and 1-methoxypyrene were downregulated. The significant changes in the identified urinary biomarkers related to gut microbiota dysbiosis are shown in the histogram (Figure 2E).

### Fecal Metabolic Profiles of Gut Microbiota Dysbiosis With and Without TACS Treatment

**FIGURE 3:**
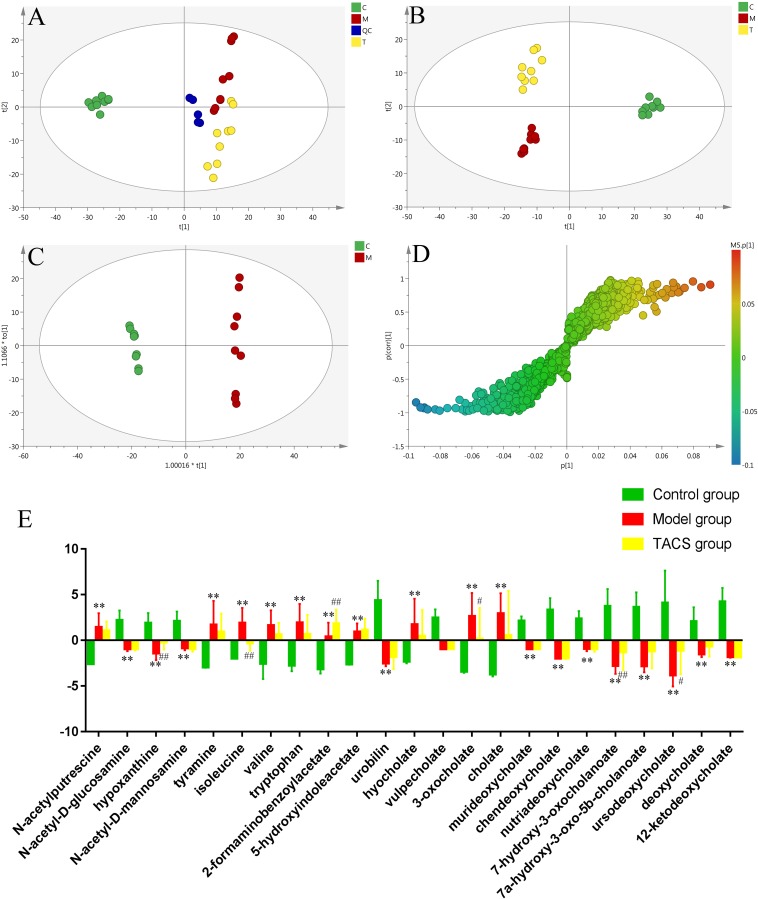
Principal component analysis score plot of fecal samples collected from different rat treatment groups in the positive mode (**A**, *R*^2^X = 0.535, *Q*^2^(cum) = 0.202). PLS-DA score plot of fecal samples collected from different rat treatment groups in the positive mode [**B**, *R*^2^X = 0.42, *R*^2^Y = 0.987, *Q*^2^(cum) = 0.917]. OPLS-DA score plot and S-plot of the control and antibiotic-only groups in the positive mode **(C,D)**. (C), control group; (M), antibiotic-only group; (T), TACS group; and (QC), quality control group. Significant changes in fecal metabolites in different groups are shown in the histogram **(E)**. (Antibiotic-only group vs. control group: ^∗^*p <* 0.05; ^∗∗^*p <* 0.01; TACS group vs. model group: ^#^*p <* 0.05; ^##^*p <* 0.01).

UPLC-Q-TOF/MS in the positive ion mode was performed to investigate the fecal samples from all three groups. The representative BPI chromatograms of each group are shown in Supplementary Figure 8. PCA and PLS-DA were carried out to discriminate between the three groups and to evaluate the metabolic patterns of the three groups. The results showed that the metabolic profile of the antibiotic-only group was different from that of the control group, while the TACS group obviously differed from the antibiotic-only group but was similar to the control group (Figure 3A,B). The results suggested that antibiotic administration induced significant metabolic changes in feces, and these changes could be modulated by TACS treatment.

### Potential Fecal Biomarkers Associated With Gut Microbiota Dysbiosis

OPLS-DA, which enhanced the established separation of the antibiotic-only and control groups, and the S-plot were employed to identify the metabolites that contributed to the clustering (Figure 3C,D). Seven-fold cross-validation was used to evaluate the predictive abilities of the constructed OPLS-DA model. Furthermore, to prevent against model over-fitting, a 200-times permutation test was performed between the control and antibiotic-only groups (Supplementary Figure 9). As revealed in Figure 3D, twenty-three significantly altered metabolites in the feces were identified as potential biomarkers. Their retention times, *m/z*, and VIP values are listed in Supplementary Table 4. The selected endogenous metabolites were characterized based on the accurate mass and the MS^E^ spectra measurements obtained *via* Q-TOF/MS and comparison of the data with literature and/or online databases. This method identified 23 metabolites found in feces as potential biomarkers of gut microbiota dysbiosis, comprising *N*-acetylputrescine, *N*-acetyl-D-glucosamine, hypoxanthine, *N*-acetyl-D-mannosamine, tyramine, isoleucine, valine, tryptophan, 2-formaminobenzoylacetate, 5-hydroxyindoleacetate, urobilin, hyocholate, vulpecholate, 3-oxocholate, cholate, murideoxycholate, chenodeoxycholate, nutriacholate, 7-hydroxy-3-oxocholanoate, 7a-hydroxy-3-oxo-5b-cholanoate, ursodeoxycholate, deoxycholate, and 12-ketodeoxycholate.

Among these metabolites, *N*-acetylputrescine, tyramine, isoleucine, valine, tryptophan, 2-formaminobenzoylacetate, 5-hydroxyindoleacetate, hyocholate, 3-oxocholate, and cholate were clearly increased in antibiotic-only rats compared to the controls, and *N*-acetyl-D-glucosamine, hypoxanthine, *N*-acetyl- D-mannosamine, urobilin, vulpecholate, murideoxycholate, chenodeoxycholate, nutriacholate, 7-hydroxy-3-oxocholanoate, 7a-hydroxy-3-oxo-5b-cholanoate, ursodeoxycholate, deoxycholate, and 12-ketodeoxycholate were significantly decreased in antibiotic-only rats compared to the controls. After TACS treatment, the levels of hypoxanthine, 2-formaminobenzoylacetate, 7-hydroxy-3-oxocholanoate, and ursodeoxycholate were upregulated compared to the levels in the controls, while isoleucine and 3-oxocholate were obviously downregulated. The significant changes in the identified fecal biomarkers related to gut microbiota dysbiosis are depicted in the histogram (Figure 3E).

### Predicted Function of Gut Microbiota

**FIGURE 4:**
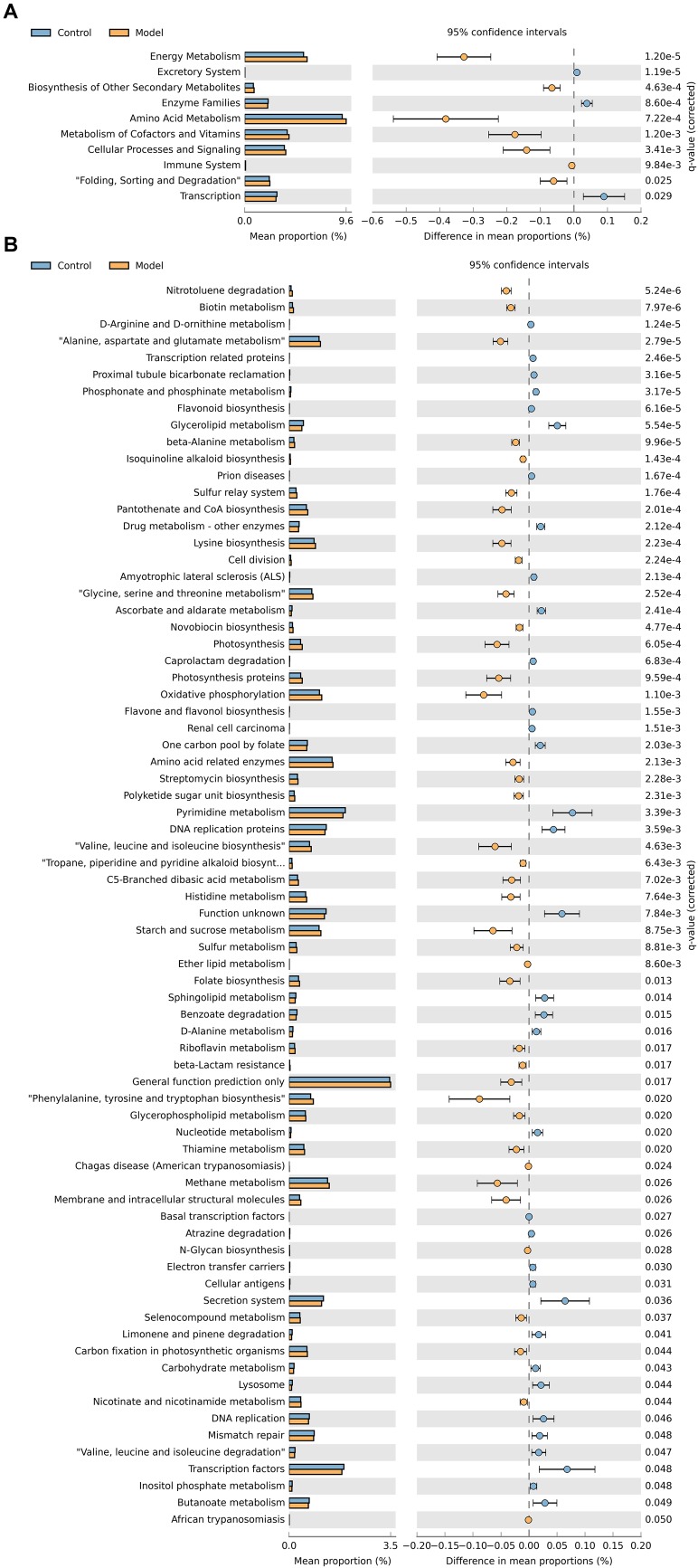
Predicted metabolic functions of cecum microbiota in the control and antibiotic-only groups. Second-level **(A)** and third-level **(B)** Kyoto Encyclopedia of Genes and Genomes (KEGG) pathways are shown in the extended error bar. *p-*values are shown on the right.

To evaluate the differences in the functional capacity of the intestinal bacterial community between the control and antibiotic-only group, PICRUSt analysis was performed to analyze the KEGG pathways related to the intestinal microbiota. As shown in Figure 4A, the second-level KEGG pathway analysis showed that energy metabolism and amino acid metabolism were enriched in the antibiotic-only group compared to the control group, while the enzyme family was decreased. Consistent with the second-level KEGG pathways results, Figure 4B shows that the antibiotic-only group had significantly elevated relative abundances of genes involved in phenylalanine, tyrosine, and tryptophan biosynthesis; glycine, serine, and threonine metabolism; amino acid–related enzyme, alanine, aspartate, and glutamate metabolism; and valine, leucine, and isoleucine biosynthesis (*p <* 0.05). Meanwhile, the abundance of genes involved in carbohydrate metabolism, nucleotide metabolism, and drug metabolism of other enzymes was decreased (*p* < 0.05).

### Correlations Between Altered Gut Microbiota and Significant Changed Metabolites in Urine and Feces Associated With Gut Microbiota Dysbiosis

**FIGURE 5:**
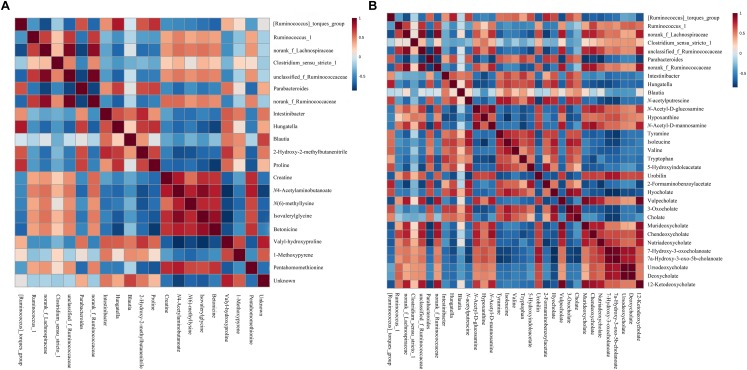
Correlation heat map showing the significant statistical correlation values (*r*) between perturbed gut microbiota genera and altered urinary **(A)** and fecal **(B)** metabolites in rats with antibiotic-induced gut microbiota dysbiosis. Red squares represent significant positive correlations (*r* > 0.5, *p* < 0.05), blue squares represent significant negative correlations (*r* < –0.5, *p* < 0.05), and white squares represent non-significant correlations.

To identify the potential link between altered gut microbiota and potential biomarkers in urine and feces involved in gut microbiota dysbiosis (*r* < -0.5 or *r* > 0.5, *p* < 0.05), we performed a Spearman’s rank-order correlation analysis. This analysis identified multiple significant associations between the perturbed gut microbiota and altered metabolites in rats with antibiotic-induced gut dysbiosis (Figure 5).

According to the correlation analysis between altered gut microbiota and potential urinary biomarkers, betonicine was positively correlated with *g__Ruminococcus_1*, *g__norank_f_Ruminococcaceae*, and *g__unclassified_f_Ruminococcaceae*. Creatine, *N*4-acetylaminobutanoate, *N*(6)-methyllysine, isovalerylglycine, and pentahomomethionine were positively correlated with *g__norank_f_Lachnospiraceae*,*g__norank_f_Ruminococcaceae*, and *g__unclassified_f_Ruminococcaceae*. Meanwhile, creatine was also positively correlated with *g__Ruminococcus_1*. 2-Hydroxy-2-methylbutanenitrile and proline were positively correlated with *g__Ruminococcus_torques_group*, *g__Hungatella*, *g__Parabacteroides*, and *g__Intestinibacter*. Valyl-hydroxyproline was positively correlated with *g__Blautia*, *g__Hungatella*, *g__Parabacteroides*, and *g__Intestinibacter*, and 1-methoxypyrene was positively correlated with *g__Blautia* and *g__Intestinibacter.*

Regarding the fecal potential biomarkers, all the elevated potential biomarkers were positively correlated with *g__Hungatella*, and all the elevated biomarkers but for valine and 2-formaminobenzoylacetate were positively correlated with*g__Parabacteroides* and *g__Intestinibacter*. *N*-acetylputrescine, isoleucine, tyramine, valine, 5-hydroxyindoleacetate, 2-formaminobenzoylacetate, hyocholate, and cholate were positively correlated with *g__ Ruminococcus_torques_group*, while isoleucine was also positively correlated with*g__Blautia*. *N*-acetyl-D-mannosamine, chenodeoxycholate, and 12-ketodeoxycholate were positively correlated to *g__Ruminococcus_1*, *g__norank_f_Lachnospiraceae*, *g__norank_f_Ruminococcaceae*, and *g__unclassified_f_Ruminococcaceae*, and ursodeoxycholate and deoxycholate were positively correlated with *g__Ruminococcus_1*. Also, *N*-acetyl-D-glucosamine, nutriadeoxycholate vulpecholate, murideoxycholate, urobilin, and 7a-hydroxy-3-oxo-5b-cholanoate were positively correlated with *g__norank_f_Lachnospiraceae*, *g__norank_f_Ruminococcaceae*, and *g__unclassified_f_Ruminococcaceae*. 7-Hydroxy-3-oxocholanoic acid was positively correlated with *g__norank_f_Lachnospiraceae*, while hypoxanthine was positively correlated with *g__Clostridium_sensu_stricto_1*.

### Key Metabolic Pathways Involved in TACS Treatment of Antibiotic-Induced Gut Microbiota Dysbiosis

The key metabolic pathways were mapped using MetaboAnalyst 3.5^7^ by integration of all identified potential biomarkers. As shown in Supplementary Figure 10, six metabolic pathways were considered to be the key pathways that may be important to antibiotic-induced gut microbiota dysbiosis (impact values > 0.01) (Wang et al., 2012). They comprised the pathways associated with valine, leucine and isoleucine biosynthesis, tryptophan metabolism, amino sugar and nucleotide sugar metabolism, arginine and proline metabolism, tyrosine metabolism, and purine metabolism. According to Supplementary Tables 3, 4, in the TACS group, the levels of *N*4-acetylaminobutanoate, betonicine, isovalerylglycine, hypoxanthine, 7-hydroxy-3-oxocholate, and ursodeoxycholate were significantly elevated compared to antibiotic-only group, while the levels of valyl-hydroxyproline, 1-methoxypyrene, isoleucine, and 3-oxocholate were decreased significantly compared to the antibiotic-only group, suggesting that TACS can regulate disorders of the most relevant metabolic pathways in the pathological process of antibiotic-induced gut microbiota dysbiosis. The microbial diversity results indicated that TACS also modulated the gut microbiota dysbiosis. Spearman’s correlation analysis showed that the 10 potential biomarkers recovered by TACS were correlated with *g__Blautia* and *g__Intestinibacter*, suggesting that the intervening effects of TACS may be achieved *via* regulation of the most relevant metabolic pathways, including branched-chain amino acid (BCAA) metabolism, bile acid metabolism, arginine and proline metabolism, and purine metabolism.

**FIGURE 6:**
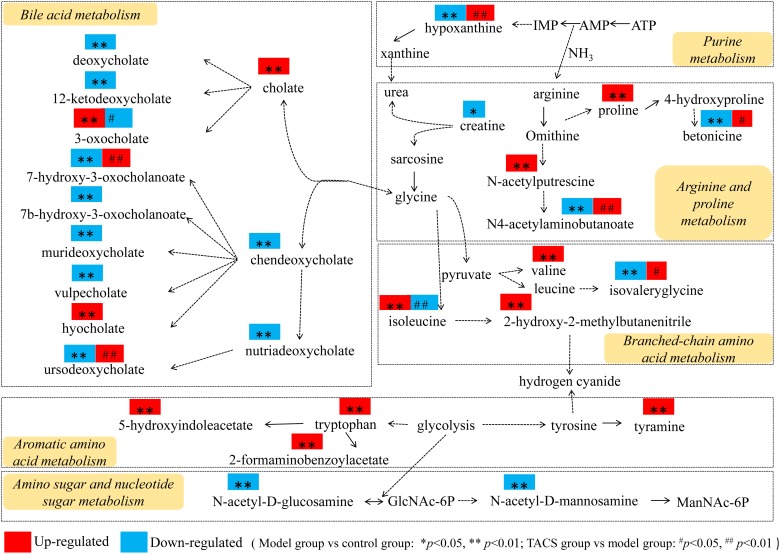
Comprehensive metabolic network of the metabolites and the major metabolic pathways in rats with antibiotic-induced gut microbiota dysbiosis.

Thus, the comprehensive metabolic network construction (using KEGG and based on the identified potential biomarkers) revealed that BCAA metabolism, bile acid metabolism, arginine and proline metabolism, purine metabolism, aromatic amino acid metabolism, and amino sugar and nucleotide sugar metabolism were the key metabolic pathways involved in the metabolic changes in rats with gut microbiota dysbiosis treated with TACS (Figure 6). However, after combining the metabolic pathway analysis with Spearman’s correlation analysis, only four metabolic pathways (BCAA metabolism, bile acid metabolism, arginine and proline metabolism, and purine metabolism) were identified as the most relevant metabolic pathways involved in the intervening effects of TACS against antibiotic-induced gut microbiota dysbiosis.

### Molecular Docking

**FIGURE 7:**
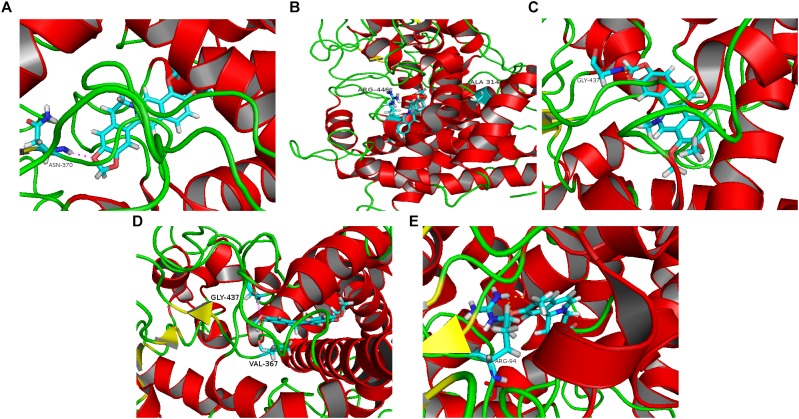
Molecular docking analysis predicting the binding sites on CYP27A1 for the five main alkaloid components of TACS: **(A)** berberine; **(B)** palmatine; **(C)** chelerythrine; **(D)** jatrorrhizine; and **(E)** dehydrocavidine.

Five alkaloid components of TACS (berberine, palmatine, chelerythrine, jatrorrhizine, and dehydrocavidine) were used in a molecular docking analysis with CYP27A1, the key enzyme in bile acid synthetic pathways. The total docking simulation scores of berberine, palmatine, chelerythrine, jatrorrhizine, and dehydrocavidine were 9.5164, 8.5358, 6.8842, 4.4558, and 4.4558, respectively. As shown in Figure 7, the binding sites of berberine, palmatine, chelerythrine, jatrorrhizine, and dehydrocavidine were ASN-370, ALA-314 and ARG-446, GLY-437, VAL-367 and GLY-437, and ARG-94, respectively.

## Discussion

This study is the first to investigate the intestinal intervening effects of TACS on rats with antibiotic-induced gut microbiota dysbiosis using 16S rRNA gene sequencing analysis combined with untargeted metabolomics. Microbial diversity analysis indicated that the rat model of gut microbiota dysbiosis was successfully established by antibiotic administration, and the dysbiosis could be improved by TACS treatment. The trends regarding *p__Firmicutes* and *p__Bacteroidetes* in the antibiotic-only group in this study were consistent with the results of previous study that also used imipenem and cilastatin sodium (Yu et al., 2017, p. 34), while they were different from other studies. The fact that different kinds of β-lactam antibiotics have different antibacterial effects might be the main reason why these differences occurred.

As shown in Table 1, antibiotic administration clearly altered the relative abundance of 10 genera, and 4 of them were restored by TACS treatment. In the TACS group, *g__Blautia* was completely restored to the control level, while incomplete restoration was found for *g__Clostridium_sensu_stricto_1*, *g__Hungatella*, and *g__Intestinibacter*. Shen et al. (2017) found that elevated *g__Blautia* may be a primary contributor to non-alcoholic fatty liver disease progression (Shen et al., 2017, p. 73), indicating that regulation of *g__Blautia* by TACS might improve health. Moreover, studies have shown that *g__Hungatella* and *g__Intestinibacter* produced acetate, which leads to activation of the microbiome–brain axis, which, in turn, promotes increased glucose-stimulated insulin secretion, increased ghrelin secretion, hyperphagia, obesity, and related sequelae (Gerritsen et al., 2014, p. 74; Kaur et al., 2014, p. 75; Perry et al., 2016, p. 76). Wlodarska et al. (2015, p. 77) found that the interaction between eugenol and *g__Clostridium_sensu_stricto_1* thickened the mucus layer and prevented intestinal infection. Therefore, the TACS-induced improvement of antibiotic-induced gut microbiota dysbiosis may have resulted in decreased acetate and increased mucus layer thickness, which may have protected the intestinal barrier and host health. Moreover, the alteration of the gut microbiota by antibiotics affects the metabolic profile of the gut microbiota. In this study, there was a higher enrichment of functions involved in energy metabolism and amino acid metabolism in the antibiotic-only group. In particular, elevated phenylalanine, tyrosine, and tryptophan biosynthesis; glycine, serine, and threonine metabolism; alanine, aspartate, and glutamate metabolism; and valine, leucine, and isoleucine biosynthesis were found in the antibiotic-only group. These metabolic pathways produced various amino acids (such as BCAAs) that are known to be connected to the risk of obesity, diabetes, and other metabolic diseases (Pietiläinen et al., 2008, p. 80).

The metabolomics results showed the different metabolic profiles of the control, antibiotic-only, and TACS groups, showing that TACS could regulate the abnormal metabolic profiles of rats with antibiotic-induced gut microbiota dysbiosis. Using OPLS-DA, 34 metabolites were screened and identified as potential biomarkers involved in the process of gut microbiota dysbiosis, including creatine, *N*(6)-methyllysine, valyl-hydroxyproline, proline, 2-hydroxy-2-methylbutanenitrile, *N*4-acetylaminobutanoate, betonicine, isovalerylglycine, 1-methoxypyrene, pentahomomethionine, *N*-acetylputrescine, *N*-acetyl-D-glucosamine, hypoxanthine, *N*-acetyl-D-mannosamine, tyramine, isoleucine, valine, tryptophan, 2-formaminobenzoylacetate, 5-hydroxyindoleacetate, urobilin, hyocholate, vulpecholate, 3-oxocholate, cholate, murideoxycholate, chenodeoxycholate, nutriacholate, 7-hydroxy-3-oxocholanoate, 7a-hydroxy-3-oxo-5b-cholanoate, ursodeoxycholate, deoxycholate, and 12-ketodeoxycholate. The changes in the metabolites suggested that valine, leucine, and isoleucine biosynthesis; tryptophan metabolism; amino sugar and nucleotide sugar metabolism; arginine and proline metabolism; tyrosine metabolism; and purine metabolism were disturbed in the antibiotic-only rats. Metabolic pathway analysis (by integration of all identified potential biomarkers with the comprehensive metabolite network constructed using a KEGG analysis) highlighted the detailed metabolic pathways involved in the intestinal intervening effects of TACS against antibiotic-induced gut microbiota dysbiosis. This indicated that TACS synergistically modulated four metabolic pathways (BCAA metabolism, bile acid metabolism, arginine and proline metabolism, purine metabolism) to modulate the gut microbiota dysbiosis. Based on the results of the metabolic pathway analysis and the predicted metabolic functions of the gut microbiota, we hypothesized that the intervening effects of TACS against gut microbiota dysbiosis mainly occurred *via* regulation of amino acid metabolism and bile acid metabolism.

### Branched-Chain Amino Acid Metabolism

Branched-chain amino acids (including leucine, valine, and isoleucine) and their metabolites can stimulate protein synthesis, mediate levels of neurotransmitter, promote insulin secretion, and decrease appetite and body adiposity by activating the mechanistic target of rapamycin (mTOR) signaling pathway. They can also be transported to the brain and influence neurotransmitter synthesis, resulting in different effects on body behavior and brain function (Nishitani et al., 2005; Pedroso et al., 2015; Zhang et al., 2017; Holecek, 2018). BCAA dysmetabolism influences many diseases. For example, patients with cirrhosis and hepatic steatosis have a decreased level of blood BCAAs (Dasarathy and Hatzoglou, 2017; Hoyles et al., 2018), while in patients with diabetes and obesity, the BCAA level is increased (Borghi et al., 1985; She et al., 2007). Studies have shown that BCAAs can be consumed and oxidized in the intestine, and BCAAs participate in bacterial metabolism and in the regulation of intestinal microbial species and diversity (Zhang et al., 2017). Leucine can increase villus height in the duodenum in order to enhance intestinal development, while isoleucine supplements can enrich *Lactobacillus* and inhibit the growth of *Escherichia coli* to improve the gut microbial population, forming a healthy gut microbial environment (Zhao et al., 2014; Sun et al., 2015).

In our previous study, the elevated level of isoleucine in the plasma of rats with CCl_4_-induced acute hepatic injury was downregulated by *Corydalis saxicola* Bunting (CS), indicating that CS might modulate BCAA metabolism by promoting protein synthesis (Liang et al., 2016). The elevated isoleucine in rats with antibiotic-induced gut dysbiosis in the present study probably occurred due to increased proteolysis and decreased diversity of BCAA-utilizing bacteria. Additionally, isoleucine was positively correlated with *g__Blautia*, which has been reported to be a contributor to non-alcoholic fatty liver disease (Shen et al., 2017). After TACS treatment, the disorders related to isoleucine and *g__Blautia* were corrected, suggesting that TACS regulates intestinal bacteria dysbiosis-induced BCAA dysmetabolism by inhibiting proteolysis and modulating gut bacteria diversity.

### Bile Acid Metabolism

The primary bile acids (cholic acid and chendeoxycholic acid) produced by the liver and metabolized into secondary bile acids by the 7alpha-dehydroxylating gut microbiota promote intestinal nutrient absorption and biliary cholesterol secretion to maintain bile acid homeostasis. The bile acids are essential for protecting the liver and other tissues and cells from cholesterol and bile acid toxicity (Ramirez-Perez et al., 2017; Chiang and Ferrell, 2018). Hepatic lipid and glucose metabolic homeostasis and energy metabolism are regulated by bile acids, which are endogenous ligands that can activate nuclear receptor farnesoid X receptor (FXRα) and membrane G protein–coupled bile acid receptor-1 (GPBAR1 or TGR5) (Lefebvre et al., 2009; Chiang, 2013). Evidence has shown (Ridlon et al., 2013) that bile acids are synthesized by classic pathways in healthy livers. When sufficient bile acids are secreted into the small intestine, they are metabolized into secondary bile acids, which maintain the stabilized gut environment and protect the gut against damage from inflammatory factor. During hepatic fibrosis, CYP7A1 expression in the classic pathway is inhibited by pro-inflammatory cytokines, which results in dysmetabolism of bile acids. The bile acid–metabolizing gut bacteria then have insufficient energy, leading to decreased bacterial abundance, which reduces the small intestine’s defense against inflammatory factors such as lipopolysaccharide (LPS), leading to inflammation-induced gut dysbiosis.

The increased levels of hyocholate, 3-oxocholate, and cholate in the antibiotic-only group showed that bile acid dysmetabolism occurred, and these bile acids were positively correlated with three increased genera (*g__Blautia, g__Parabacteroides*, and *g__Intestinibacter*). Our results were consistent with the previous study. However, the increase in this study of *g__Blautia*, a kind of 7alpha-dehydroxylating bacteria, exhibited the opposite trend compared to the decrease in the secondary bile acids. According to previous studies, increased *g__Blautia* in feces has been found in Hashimoto’s thyroiditis, hemorrhagic diarrhea, and non-alcoholic liver diseases (Shen et al., 2017; Zeineldin et al., 2018; Zhao F. et al., 2018). Therefore, we hypothesized that the elevated level of *g__Blautia* was probably influenced by interaction between bile acid dysmetabolism and inflammation. Research has shown that the gut microbial composition of rats fed a high-fat diet was influenced by abnormal bile acid metabolism (Zheng et al., 2017). In our TACS group, the *g__Blautia* was restored, and the levels of certain secondary bile acids were also restored, which indicated that TACS may regulate the composition and amount of gut bacteria by modulating bile acid dysmetabolisms and inflammation, making it possible to improve gut self-stabilization.

### Arginine and Proline Metabolism

*N*-acetylputrescine, which is metabolized by gut bacteria, is a polyamine. Polyamines play a vital role in animal, plant, and bacteria growth by acting as growth factors, which involves binding to nucleic acids and promoting protein and nucleic acid synthesis; if polyamine biosynthesis is disrupted, cell growth is inhibited (Matsumoto et al., 2012; Sugiyama et al., 2016). Recent studies have shown that polyamines have anti-inflammatory effects and promote autophagy, and they can also be absorbed from the intestinal lumen to repair and maintain the intestinal mucosal barrier (Eisenberg et al., 2009; Timmons et al., 2012; Kibe et al., 2014). Meanwhile, the gut bacteria are involved in modulating intestinal luminal polyamines levels (Matsumoto and Benno, 2007). Betonicine (also named hydroxy stachydrine), a derivative of proline metabolites, inhibits increased capillary permeability and inflammatory exudation induced by acute inflammation *via* the arachidonate pathway, decreasing the levels of pro-inflammatory factors and decreasing cell permeability (Wang and Wang, 2012). Additionally, betonicine is known to be an osmoprotectant and cryoprotectant; it can protect bacteria from extreme temperature and high-salt environments *in vitro* and enhance bacterial growth (Bayles and Wilkinson, 2000; Bashir et al., 2014).

After antibiotic administration in the antibiotic-only group, the elevated *N*-acetylputrescine in feces may have been caused by both the enhanced polyamine catabolism due to the increased proteolysis and the intestinal mucosa injury caused by the LPS released by the damaged gut bacteria, which leads to the malabsorption of intestinal luminal polyamines. The level of proline, which was positively correlated with *g__Parabacteroides* in the antibiotic-only group, was increased. This may be the result of enriched proteolysis and inflammation caused by the antibiotics. Together, the disorder of arginine and proline metabolism and intestinal bacteria dysbiosis may have influenced the inhibition of enzymatic activity related to proline catabolism, leading to decreased levels of *N*4-acetylaminobutanoate and betonicine, both of which were negatively correlated with the inflammation-relevant genus *g__Blautia*. Nevertheless, the fact that this disorder could be ameliorated by TACS treatment suggested that TACS could correct the perturbation of arginine and proline metabolism by inhibiting proteolysis and regulating the enzymatic activity of certain reactions, resulting in modulation of the gut microbiota dysbiosis. Furthermore, the elevation of betonicine plays a protective role in the permeability of the intestinal mucosa, reducing inflammation.

### Purine Metabolism

Hypoxanthine is produced during purine catabolism. According to recent research, hypoxanthine improves hepatic pathological conditions and reduces the absorption of fat by inhibiting the activity of superoxide dismutase (SOD) and malondialdehyde (MDA), which demonstrates that hypoxanthine could help to reduce fat deposition (Xiaoyu et al., 2016). The relationship between hyperuricemia and the gut bacteria composition has already been studied, and it was found that a high-purine diet induced structural shifts in the gut microbiota and hyperuricemia in quails; this led to elevated LPS and increased xanthine oxidase (XO) activity, resulting in decreased hypoxanthine (Huang et al., 2015). The reduced hypoxanthine in the feces of the rats with antibiotic-induced gut dysbiosis was probably due to the increased intestinal LPS released by the dying bacteria. XO activity may have been increased, leading to a lower level of hypoxanthine and an increased level of uric acid. Additionally, evidence shows that XO activity influences superoxide radical production, which leads to oxidative stress that may induce or aggravate hepatic diseases (Masarone et al., 2018). According to the correlation analysis, the inflammation-relevant genera were negatively correlated with hypoxanthine, indicating that TACS recovered the gut microbiome diversity and repressed XO activity by improving the abnormal purine metabolism.

### Aromatic Amino Acid Metabolism

Tryptophan metabolites, which are metabolites that are strongly associated with gut bacteria, can be metabolized into indole by tryptophanase from gut bacteria, particularly *Escherichia coli* (Botsford and Demoss, 1972). Antibiotic treatment rapidly decreased the gut bacteria community, and the amount of tryptophanase also decreased, leading to disruption of the tryptophan–indole pathway and elevated fecal tryptophan. After TACS treatment, the level of tryptophan was slightly reduced, which indicates that TACS mildly regulated the disordered tryptophan metabolism, increasing the levels of some of the gut bacteria. This led to the regulation of the disordered tryptophan metabolism, improving the abnormal metabolic profile.

### Amino Sugar and Nucleotide Sugar Metabolism

*N*-acetyl-D-glucosamine, which is related to amino sugar and nucleotide sugar metabolism, recovers intestinal epithelial cells, reduces permeability (thereby stabilizing the intestine barrier); inhibits intestinal bacteria from entering the circulation; and has antibacterial, antitumor, and antioxidant effects (Krenek et al., 2007; Ying et al., 2011). Antibiotic treatment disrupts the balance of the rat gut microbial environment, causing gut bacteria dysbiosis and metabolism disorders and resulting in a lower level of *N*-acetyl-D-glucosamine. This weakens the stability of the gut barrier and increases its permeability. After TACS treatment, *N*-acetyl-D-glucosamine was mildly upregulated, demonstrating that TACS regulates the disordered amino sugar and nucleotide sugar metabolism. The effects of TACS maintained the function of the intestinal epithelial cells, reduced the permeability (thereby stabilizing the intestinal barrier), and improved the gut microbial environment, improving the abnormal metabolic profile.

### Molecular Docking

Gut microbiota abnormalities, which were associated with changes in bile acids, were demonstrated. Of the 34 altered metabolites, 12 were bile acids, which indicated that the bile acid pathway could be one of the most relevant metabolic pathways underlying dysbiosis and the therapeutic effects of TACS in antibiotic-induced gut microbiota dysbiosis. Therefore, the dysbiosis in our study might have been caused by perturbations in the bile acid metabolic pathway. We hypothesized that the intervening effects of TACS on gut microbiota dysbiosis may act by influencing bile acid synthesis and metabolism. Thus, to simulate how ligands act in a complex molecular network, we performed a molecular docking analysis using the main alkaloid components of TACS as ligands, with CYP27A1 (PDB-ID: 1mfx) as the target protein, which is a key enzyme in the bile acid synthesis pathway.

There are three alkaloid components of TACS that can regulate gut microbiota dysbiosis (Deng et al., 2009; Zhou et al., 2017; Kokoska et al., 2018). We performed a molecular docking analysis to explore the alkaloid components that affect the key enzyme of the bile acid metabolism pathway, CYP27A1. The results showed that the docking scores of the five main alkaloid components, berberine, palmatine, chelerythrine, jatrorrhizine, and dehydrocavidine, from high to low, were as follows: 9.5164, 8.5358, 6.8842, 4.4558, and 4.4558. The binding sites of berberine, palmatine, chelerythrine, jatrorrhizine, and dehydrocavidine were ASN-370, ALA-314 and ARG-446, GLY-437, VAL-367 and GLY-437, and ARG-94, respectively. According to these results, we concluded that berberine (9.5164), palmatine (8.5358), and chelerythrine (6.8842) might be the major active compounds for TACS in reducing antibiotic-induced gut microbiota dysbiosis by influencing CYP27A1 during bile acid synthesis. Recently, a study by Satchidananda Panda’s group (Zarrinpar et al., 2018) found that gut microbiome depletion using antibiotics led to decreased bile acids. This reduction upregulated the expression of FXR and bile acid transport protein, and increased bile acid transport into the blood, resulting in increased bile acid synthesis in the liver, probably by increasing CYP7A1 expression. The different findings in our study may be caused by the therapeutic drug used (TACS) and the different antibiotic regimen. This is the first study to find that alkaloid components of TACS might modulate bile acid metabolism by affecting CYP27A1. Further research still needs to be carried out to investigate the mode of action of these components regarding their intervening effects in rats with antibiotic-induced gut microbiota dysbiosis.

This research aimed to explore the intervening effects of TACS against antibiotic-induced gut microbiota dysbiosis in rats. We explored the mechanism of these alkaloid components regarding the prevention of gut microbiota dysbiosis using sequencing combined with metabolomics. We focused on bile acid metabolism, which might be the main metabolic pathway, and identified five alkaloid components of TACS that may affect bile acid metabolism, leading to further influences on host co-metabolism and gut microbiota dysbiosis. However, whether these components could exactly modulate the bile acid pathway *via* the key enzyme of the bile acid synthetic pathways (CYP27A1) remains unknown. This is what we plan to investigate further in our next study.

## Conclusion

In this study, for the first time, a 16S rRNA gene sequencing analysis combined with a UPLC-Q-TOF/MS-based metabolomics approach was used to study the exact mechanism underlying the intervening effects of TACS against gut microbiota dysbiosis. The cecum microbiota composition in the antibiotic-induced gut microbiota dysbiosis group was clearly distinguishable from that of the control group. The predicted metabolic functions of the gut microbiota using PICRUSt showed abnormal amino acid metabolism in the antibiotic-only group, indicating that the antibiotic treatment induced amino acid metabolic dysbiosis in the microbiota, which led to disturbed host metabolism. The metabolomics results showed that TACS intervenes with the antibiotic-induced abnormal metabolic changes by modulating BCAA metabolism, bile acid metabolism, arginine and proline metabolism, and purine metabolism. The molecular docking results showed that three alkaloid components of TACS could bind well to CYP27A1. This suggested that TACS might act by affecting the key enzyme of the bile acid metabolic pathway (CYP27A1). Taken together, these results indicated that the UPLC-Q-TOF/MS-based and 16S rRNA gene sequencing approach is a useful and reliable method for clarifying the intervening effects of TACS on the host co-metabolism and intestinal microbiome in rats with antibiotic-induced gut microbiota dysbiosis. This study presents new insights for the discovery of effective drugs and the best therapeutic approaches. However, whether the TACS components could exactly modulate the bile acid pathway *via* the key enzyme of the bile acid synthetic pathways still needs further investigation.

## Ethics Statement

This study was approved and conducted by the Ethics Committee for Animal Experimental of Guangxi Medical University (No. 201709003).

## Author Contributions

XiL, HuZ, RL, and ZS conceived and designed the experiments. XiL, JW, and HS wrote the manuscript. HH, FW, YL, MD, and DL performed the metabolomics experiments. XuL, HoZ, and HG performed the 16S rRNA gene sequencing experiments. YM, CY, and BC analyzed the data. All authors reviewed the manuscript.

## Conflict of Interest Statement

The authors declare that the research was conducted in the absence of any commercial or financial relationships that could be construed as a potential conflict of interest.

## Footnotes

1 http://www.hmdb.ca/

2 http://www.massbank.jp/

3 http://metlin.scripps.edu

4 http://www.genome.ad.jp/kegg/

5 http://www.metaboanalyst.ca/

6 http://www.rcsb.org/

7 http://www.metaboanalyst.ca/
