# Supplementary material for: Intervening Effects of Total Alkaloids of Corydalis saxicola Bunting on Rats With Antibiotic-Induced Gut Microbiota Dysbiosis Based on 16S rRNA Gene Sequencing and Untargeted Metabolomics Analyses
Source: Front Microbiol. 2019 May 31;10:1151. doi: 10.3389/fmicb.2019.01151 (PMC6555270; doi:10.3389/fmicb.2019.01151)
Supplement: Supplementary file 1 [file Table_1.DOC]

Supplementary materials

1 Method

1.1 The characterization of chemical profile of TACS by UPLC-Q-TOF/MS

1.1.1 Chemicals

Pure water was from a manufacture of ultrapure water system (Chengdu Yue Chun Technology Co., Ltd; Chengdu, China). Formic acid was obtained from ChengDu KeLong Chemical Company (Chengdu, China). Acetonitrile (HPLC grade) was purchased from Thermo Fisher Scientific Inc. (Shanghai, China). The reference standards of Berberine, Palmatine, Chelerythrine, Epiberberine and Jatrorhizine were purchased from Chengdu Must Bio-Technology Co., Ltd (Chengdu, China), and the batch number were MUST-16111115, MUST-17022604, MUST-14062313, MUST-17072011 and MSUT-17041801 respectively. Dehydrocavidine was purchased from National Institute for the Control of Pharmaceutical and Biological Products, and the batch number was 11667-200401.

1.1.2 Extraction of TACS

The herbs of CS were purchased from Nanning Shengyuantang Chinese herbal medicine Co., Ltd (Nanning, China), and authenticated by associate professor Changming Mo of Guangxi Botanical Garden of Medicinal Plants. The herbs were cleaned, dried and cut into small pieces. The extraction process of TACS was shown in Supplementary Figure 1.


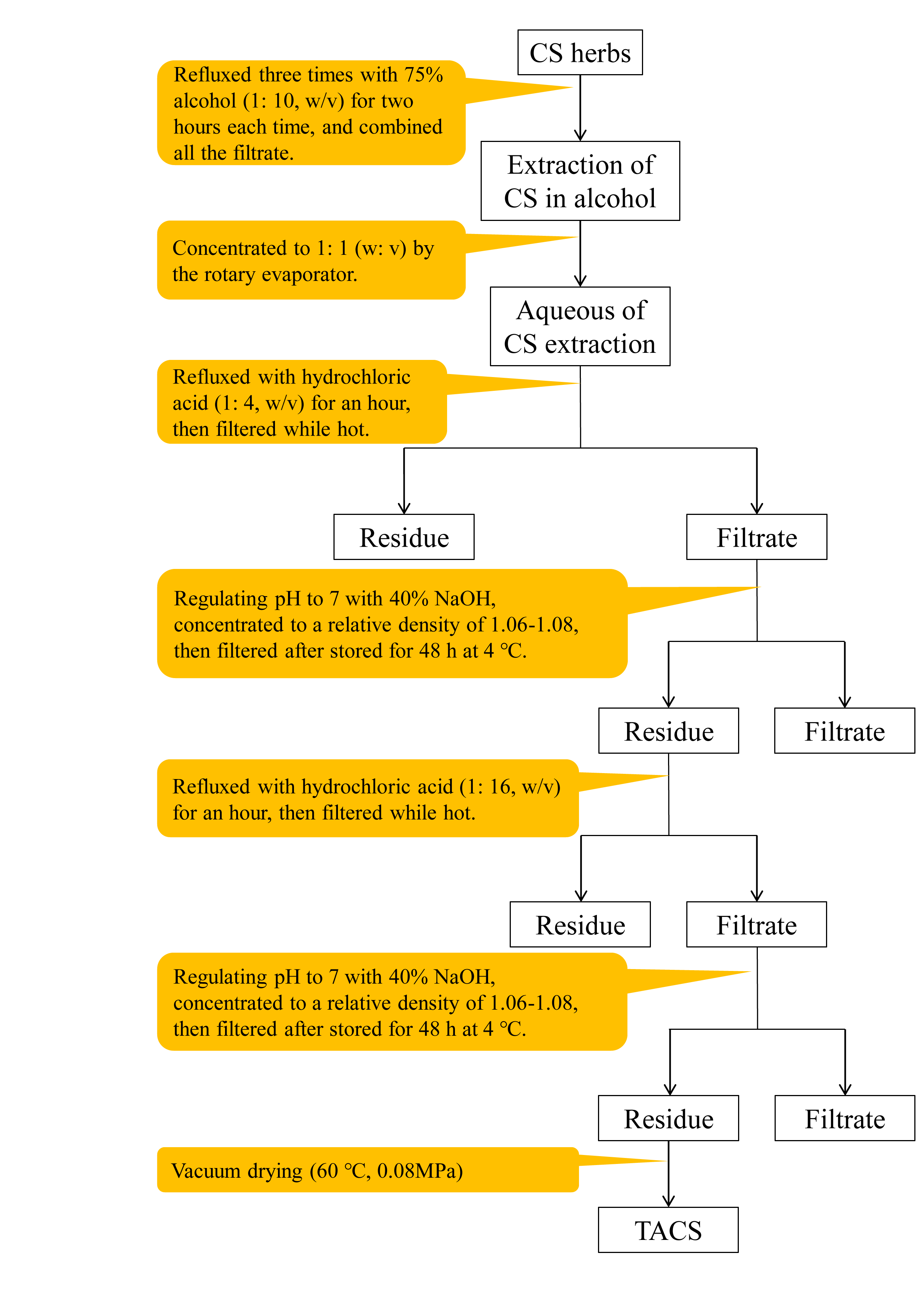


**Supplementary Figure 1.** The flow diagram of the extraction process of TACS.

1.1.3 Sample preparation

Take TACS stock powder (1.250mg) in a 2.5 ml volumetric flask and filled up to the final volume with methanol. After ultrasonic treatment with 30min, the obtained mixed solution was filtered by a syringe filter (0.22 µm), and finally stored away from light at 4 ℃.

1.1.4 Preparation of standard solutions

The mixed standard solutions containing six compounds i.e. epiberberine, jatrorhizine, dehydrocavidine, palmatine, berberine and chelerythrine were prepared by dissolving the standards in methanol to final concentration of 0.80, 0.74, 0.69, 0.76, 0.64 and 0.58 and 0.91mg/ml respectively. The solution was stored away from light at 4 ℃.

1.1.5 UPLC-Q-TOF/MS Conditions

Chromatographic analysis was performed on an Acquity UPLC HSS T3 column (100 mm × 2.1 mm, 1.8 μm) by Waters ACQUITY UPLC system (Waters Corp. Milford, USA). The columns were maintained at 40 °C and eluted at a flow rate of 0.50 mL/min. The mobile phase was composed of water (A) and acetonitrile (B) each containing 0.1% formic acid. The gradient program for urine samples was optimized as follows: 0–1.5 min, 10% B to 25% B; 1.5–3.5 min, 25% B to 30% B; 3.5–4.5 min, 30% B to 35% B; 4.5–6 min, 35% B to 60% B, 6–7.5 min, 60% B to 95% B; 7.5–8 min, 95% B to 10% B, 8–10 min, washing with 10% B. The injection volume was 0.1 μL.

The mass spectrometry with an electrospray ionization source operating in positive ion mode was performed on Waters definition accurate mass quadrupole time-of-flight (Q-TOF) XevoG2-S mass spectrometer (Waters MS Technologies, UK). The parameters were set as below: briefly, capillary voltage, 2.8 kV; sample and extraction cone voltage, 40 V and 4.0 V; desolvation gas rate and temperature, 700 L/h and 350 °C; cone gas rate, 20 L/h; source temperature, 100 °C; scan time and inter scan delay, 0.2 and 0.02 s. Leucine-enkephalin was used as the lockmass in all analyses ([M + H]^+^ = 556.2771) at a concentration of 0.5 μg/mL with a flow rate of 5 μL/min. Data was collected in centroid mode from *m/z* 100 to *m/z* 1200.

1.2 16S rRNA gene sequencing and microbial diversity analysis

Microbial DNA was extracted from cecum samples by the E.Z.N.A. ® soil DNA Kit (Omega Bio-tek, Norcross, GA, U.S.) The final DNA concentration and purification were determined by NanoDrop 2000 UV-vis spectrophotometer (Thermo Scientific, Wilmington, USA), and we used 1% agarose gel electrophoresis to check the DNA quality. The V3-V4 hyper-variable regions of the bacterial 16S rRNA gene were amplified with primers 338F (5’-ACTCCTACGGGAGGCAGCAG-3’) and 806R (5’-GGACTACHVGGGTWTCTAAT-3’) by Thermocycler PCR system (Gene Amp 9700, ABI, USA).

The PCR reactions were conducted as the following steps, 3 min of DNA denaturation at 95 °C, 27 cycles of 30 s at 95 °C, 30s for annealing at 55 °C, and 45s for elongation at 72 °C, and a final extension at 72 °C for 10 min. PCR reactions were performed in triplicate 20 μL mixture containing 4 μL of 5 × FastPfu Buffer, 2 μL of 2.5 mM dNTPs, 0.8 μL of each primer (5 μM), 0.4 μL of FastPfu Polymerase and 10 ng of template DNA. The PCR products were extracted from a 2% agarose gel and further purified using the AxyPrep DNA Gel Extraction Kit (Axygen Biosciences, Union City, CA, USA) and sequenced (2 × 300) on an Illumina MiSeq platform (Illumina, San Diego,USA).

Raw fastq files were demultiplexed, quality-filtered by Trimmomatic and merged by FLASH with the following criteria: (i) The reads were truncated at any site receiving an average quality score <20 over a 50 bp sliding window. (ii) Primers were exactly matched allowing 2 nucleotide mismatching, and reads containing ambiguous bases were removed. (iii) Sequences whose overlap longer than 10 bp were merged according to their overlap sequence. We clustered representative sequences into Operational taxonomic units (OTUs) with an open-reference approach at 97% similarity using UPARSE, (version 7.1 <http://drive5.com/uparse/>) identified and removed chimeric sequences by UCHIME. The taxonomy of each 16S rRNA gene sequence was analyzed by RDP Classifier algorithm against the Silva (SSU123) 16S rRNA database using confidence threshold of 70%. Statistical analysis of Bray-Curtis dissimilarities was calculated using the relative abundance of bacterial genera in R package (version 3.2.1) and the adonis function in the R package ‘vegan’.

2 Results

1.2.1 The qualitative identification of TACS by UPLC-Q-TOF/MS

The characterization of chemical profile of TACS was shown in Supplementary Figure 2. By comparison of retention time and *m/z*, alkaloids components in TACS were identified as epiberberine, jatrorhizine, dehydrocavidine, palmatine, berberine and chelerythrine (Supplementary Figure 3 and Table 1).


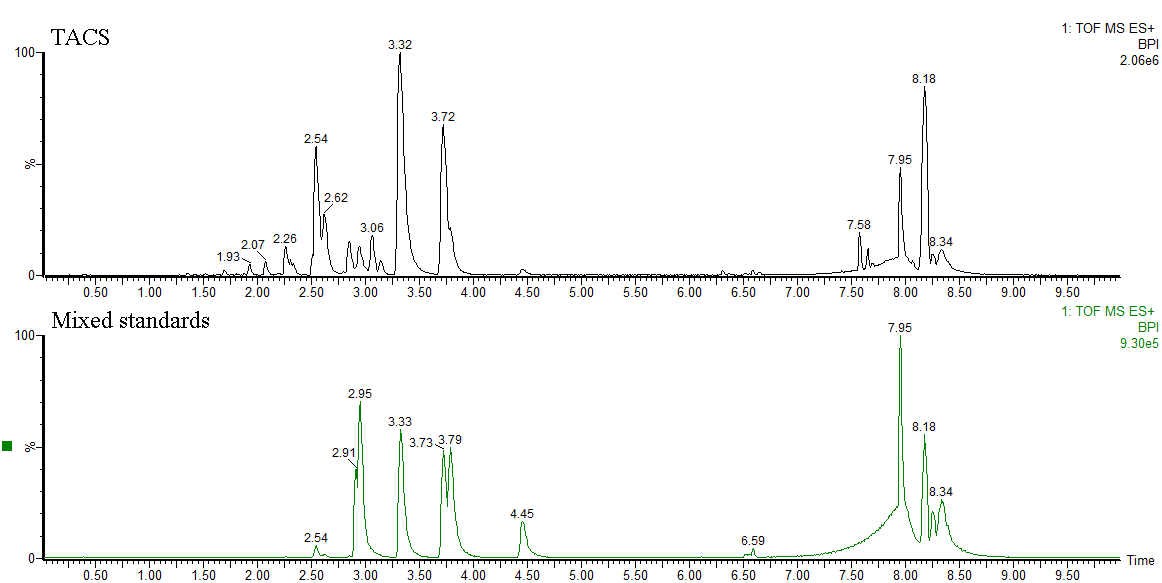


**Supplementary Figure 2.** BPI chromatograms of TACS and mixed standard.


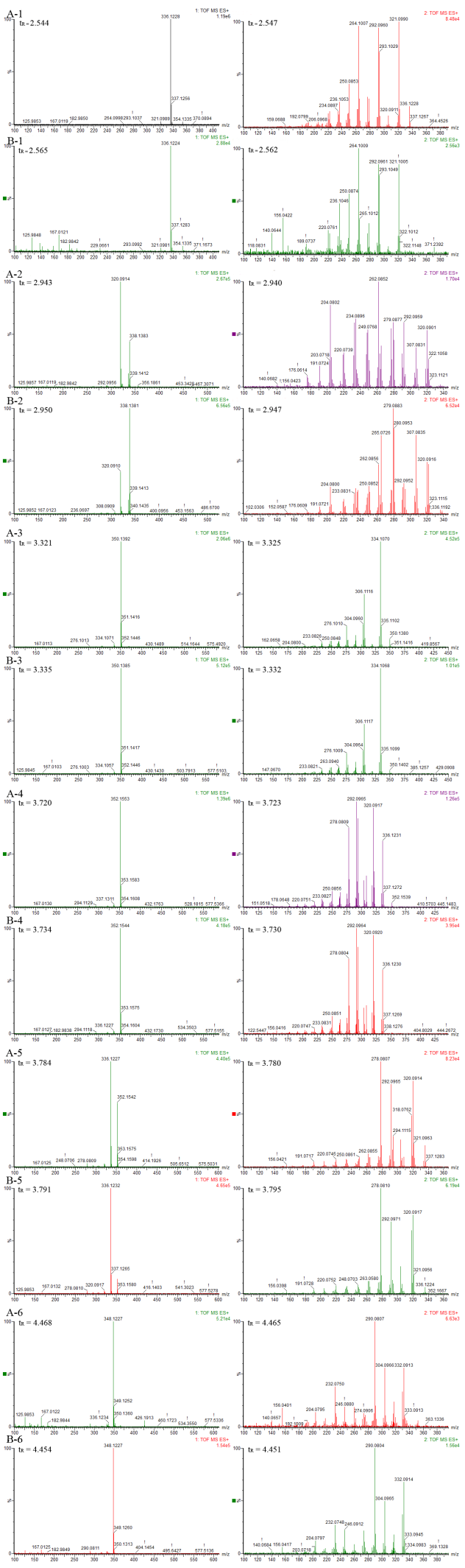


**Supplementary Figure 3.** Corresponding mass spectrums at the same retention time of TACS (A) and reference standards (B). 1 to 6 represents epiberberine, jatrorhizine, dehydrocavidine, palmatine, berberine and chelerythrine in turn


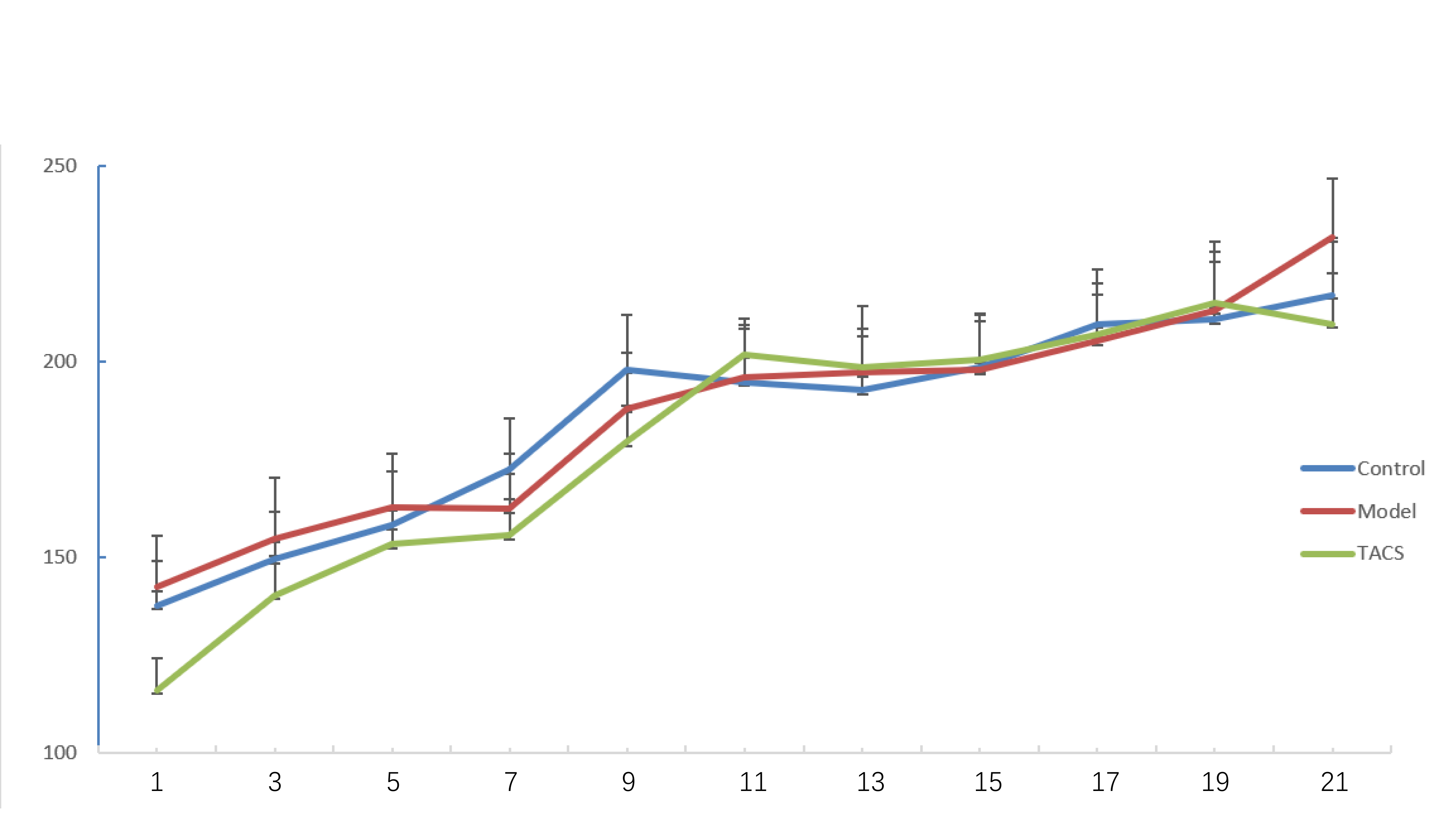


**Supplementary Figure 4.** Body weight of rats in each group.


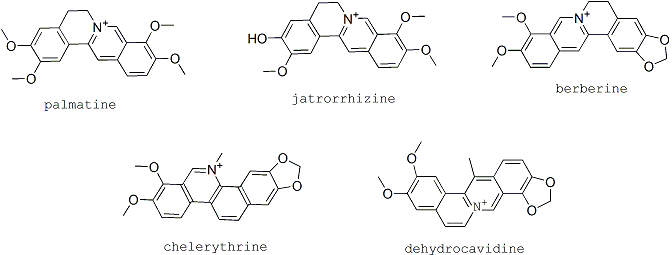


**Supplementary Figure 5.** Chemical structures of five alkaloid components of TACS which were performed molecular docking with CYP27A1.


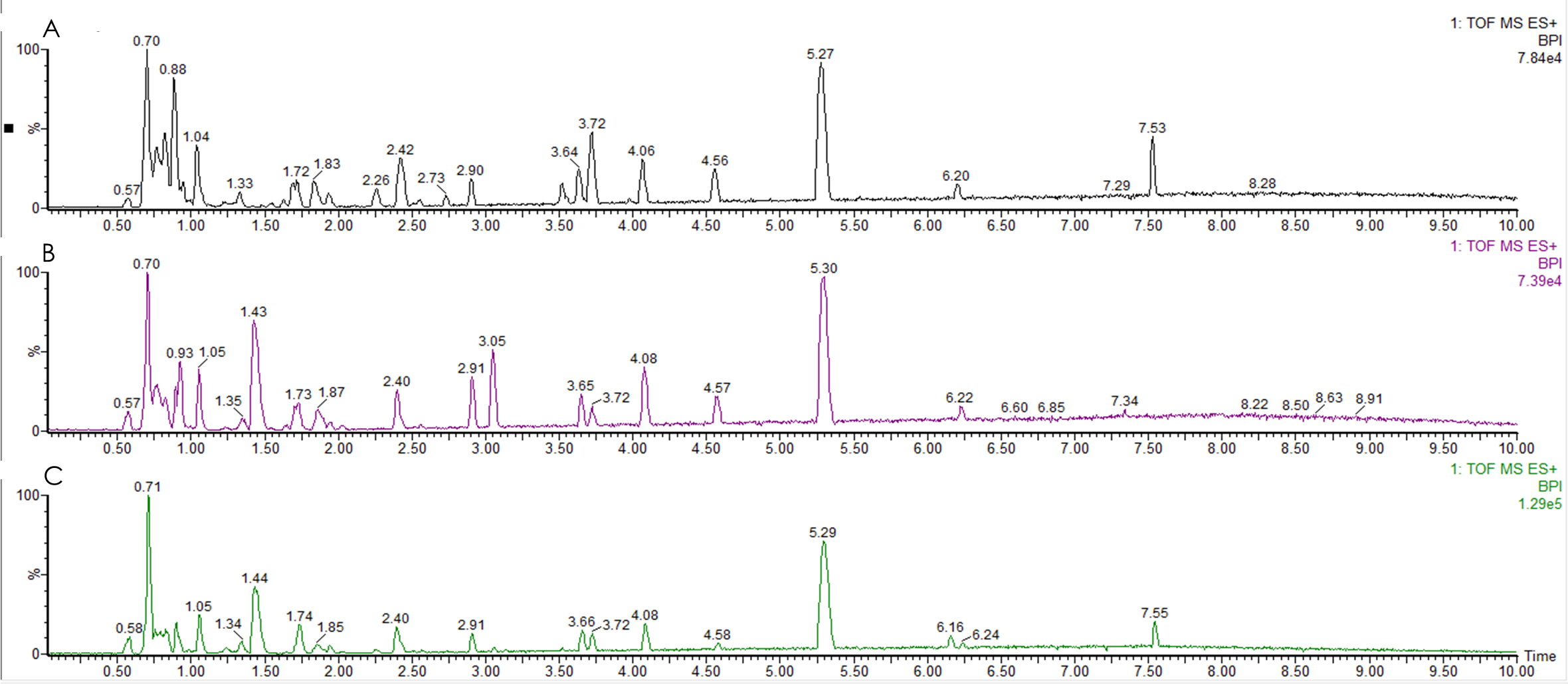


**Supplementary Figure 6.** Typical UPLC-Q-TOF/MS base peak intensity (BPI) chromatograms of urinary samples in positive ion mode. (A) control group, (B) model group, (C) TACS group.

**
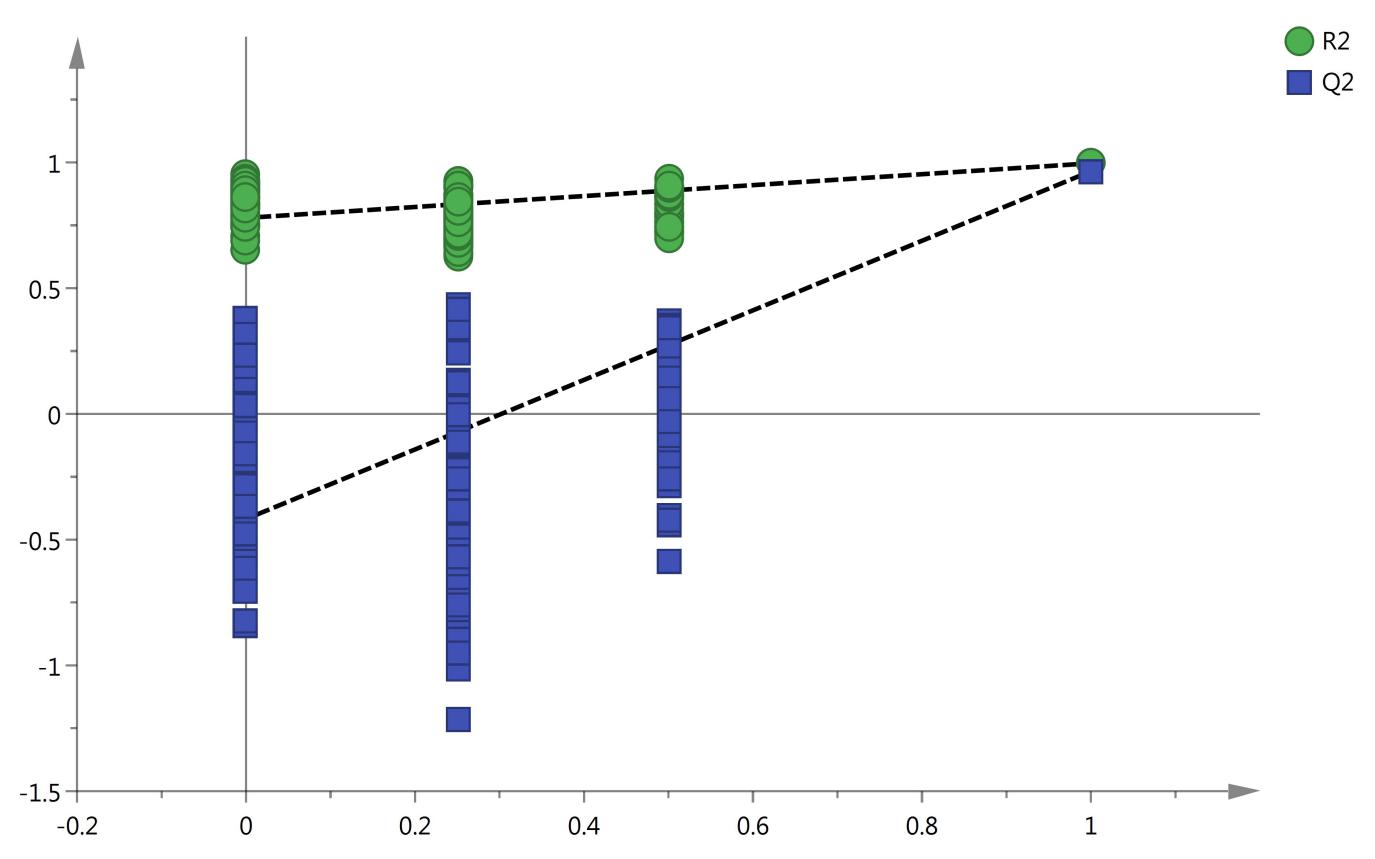
**

**Supplementary Figure 7.** The corresponding validation plots based on 200-times permutation test demonstrated the robustness of the OPLS-DA models of urinary samples between control and model groups（R^2^ = (0.0, 0.0.779); Q^2^ = (0.0, 0.-0.418)）

**
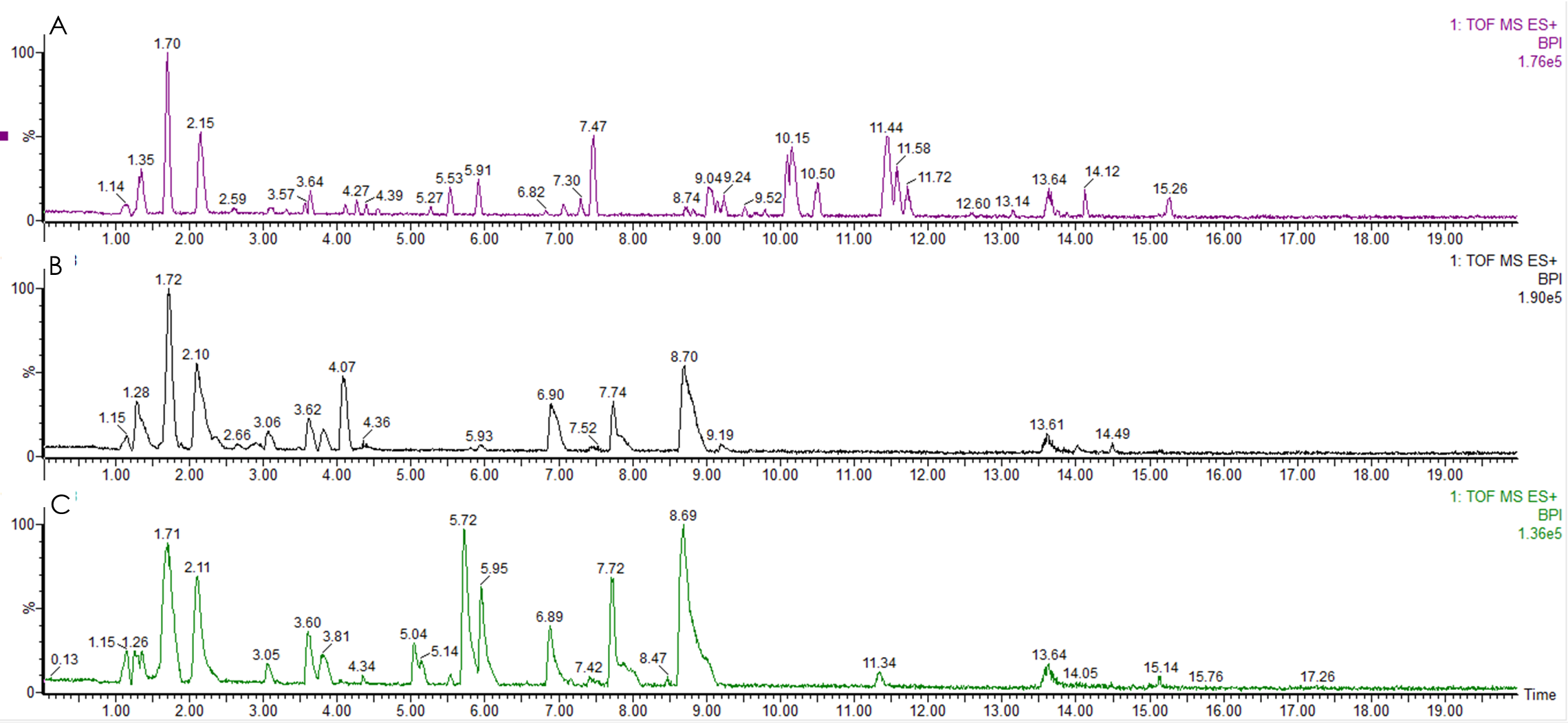
**

**Supplementary Figure 8.** Typical UPLC-Q-TOF/MS base peak intensity (BPI) chromatograms of fecal samples in positive ion mode. (A) control group, (B) model group, (C) TACS group.

**
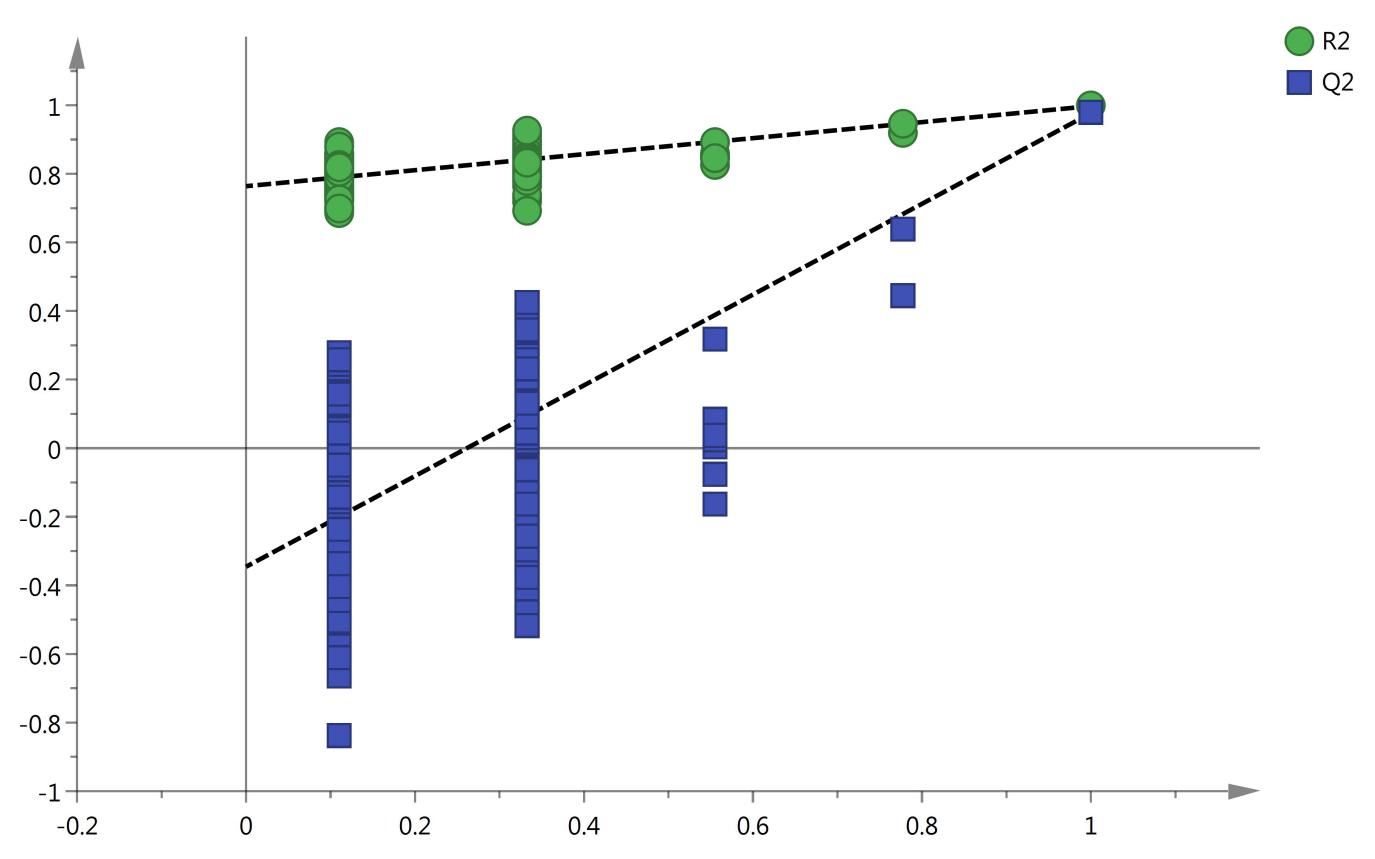
**

**Supplementary Figure 9.** The corresponding validation plots based on 200 times permutation test demonstrated the robustness of the OPLS-DA models of fecal samples between control and model groups. （R^2^ = (0.0, 0.0.764); Q^2^ = (0.0, 0.-0.346)

**
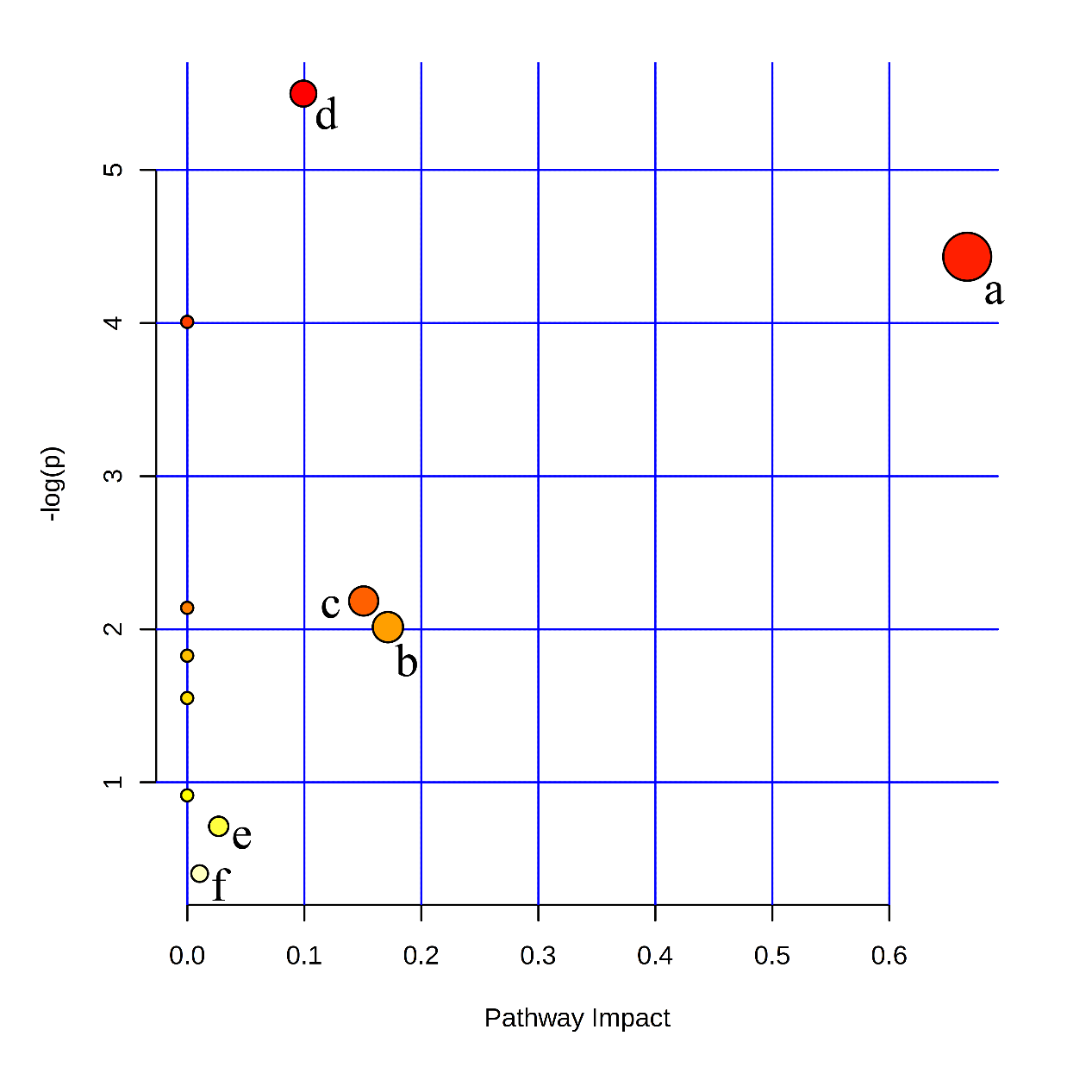
**

**Supplementary Figure 10** Summary of pathway analysis with MetaboAnalyst. (a) Valine, leucine and isoleucine biosynthesis; (b) Tryptophan metabolism; (c) Amino sugar and nucleotide sugar metabolism; (d) Arginine and proline metabolism; (e) Tyrosine metabolism; (f) Purine metabolism;

**Supplementary Table 1.** The retention time and *m/z* of epiberberine, jatrorhizine, dehydrocavidine, palmatine, berberine and chelerythrine in TACS and reference standards.

|  | t_R_/min | | *m/z* | |
| --- | --- | --- | --- | --- |
|  | TACS | Standards | TACS | Standards |
| Epiberberine | 2.54 | 2.56 | 336.1228→321.0990, 292.0960, 264.1007 | 336.1224→321.0988, 292.0961, 264.0967 |
| Jatrorrhizine | 2.94 | 2.95 | 338.1383→320.0901, 307.0831, 292.0959, 279.0877, 262.0852, 204.0802 | 338.1381→320.0916, 307.0835, 292.0952, 279.0883, 262.0856, 204.0800 |
| Dehydrocavidine | 3.32 | 3.33 | 350.1392→334.1070, 306.1116, 276.1010, 233.0826 | 350.1385→334.1068, 306.1117, 276.1009, 233.0821 |
| Palmatine | 3.72 | 3.73 | 352.1553→336.1231, 320.0917, 292.0965, 278.0809, 250.0856 | 352.1544→336.1230, 320.0920, 292.0964, 278.0804, 250.0851 |
| Berberine | 3.78 | 3.79 | 336.1227→320.0914, 292.0965, 278.0807 | 336.1232→320.0917, 292.0917, 278.0810 |
| Chelerythrine | 4.46 | 4.45 | 348.1227→332.0913, 304.0966, 290.0807, 232.0750 | 348.1227→332.0914, 304.0965, 290.0804, 232.0748 |

**Supplementary Table 2.** The reproducibility of UPLC-Q-TOF/MS method validation under the positive ion mode using QC samples (urine samples: A; feces samples: B).

A:

| No. | RT | *m/z* | repeatability | |
| --- | --- | --- | --- | --- |
|  |  |  | RSD (%) RT | RSD (%) *m/z* |
| 1 | 1.37 | 132.1033 | 0.9997 | 0.0002 |
| 2 | 1.70 | 132.1027 | 1.3614 | 0.0003 |
| 3 | 2.11 | 166.0869 | 1.2135 | 0.0001 |
| 4 | 3.08 | 205.0975 | 0.9053 | 0.0002 |
| 5 | 3.63 | 208.0611 | 0.6385 | 0.0001 |
| 6 | 7.74 | 424.3057 | 0.3713 | 0.0002 |
| 7 | 8.70 | 426.3214 | 0.4239 | 0.0001 |
| 8 | 5.91 | 595.3232 | 0.3325 | 0.0002 |
| 9 | 6.90 | 426.3232 | 0.4457 | 0.0002 |
| 10 | 10.10 | 408.3116 | 0.4144 | 0.0003 |

B:

| No. | RT | *m/z* | Repeatability | |
| --- | --- | --- | --- | --- |
|  |  |  | RSD (%) RT | RSD (%) *m/z* |
| 1 | 0.71 | 114.068 | 0.5764 | 0.0002 |
| 2 | 1.44 | 116.0721 | 0.2838 | 0.0003 |
| 3 | 2.40 | 100.0774 | 0.0000 | 0.0004 |
| 4 | 2.91 | 220.1193 | 0.2588 | 0.0002 |
| 5 | 3.65 | 206.0462 | 0.5096 | 0.0002 |
| 6 | 4.09 | 190.0513 | 0.4022 | 0.0003 |
| 7 | 5.29 | 194.0824 | 0.3467 | 0.0001 |
| 8 | 6.24 | 233.0933 | 0.4262 | 0.0001 |
| 9 | 7.55 | 220.1381 | 0.1082 | 0.0001 |
| 10 | 4.57 | 180.0667 | 0.3504 | 0.0001 |

**Supplementary Table 3. The potential biomarkers of urine in antibiotics-induced gut microbiota dysbiosis rats detected by UPLC-Q-TOF/MS. (*: *p* ＜0.05；**: *p* ＜ 0.01)**

| **No.** | **RT** | ***m/z*** | **VIP** | **MS/MS** | **Metabolites** | **Formula** | **Adduct ion** | **Pathway** | **M ：C** | **T : M** |
| --- | --- | --- | --- | --- | --- | --- | --- | --- | --- | --- |
| U1 | 0.71 | 132.0781 | 4.01894 | 111.0201;132.0781 | Creatine | C_4_H_9_N_3_O_2_ | [M+H]^+^ | Arginine and proline metabolism | ↓* | ↑ |
| U2 | 0.89 | 143.1194 | 6.83335 | 126.0926; 143.1190; 161.1268 | N(6)-Methyllysine | C_7_H_16_N_2_O_2_ | [M+H-H_2_O]^+^ | —— | ↓** | ↑* |
| U3 | 1.44 | 231.1350 | 5.54855 | 195.1127;213.1242;231.1343 | Valyl-hydroxyproline | C_10_H_19_N_2_O_4_ | [M+H]^+^ | Protein catabolism | ↑** | ↓** |
| U4 | 1.45 | 116.0722 | 8.44985 | 70.0675; 98.0615; 99.0455;116.0720 | Proline | C_5_H_9_NO_2_ | [M+H]^+^ | Arginine and proline metabolism | ↑** | - |
| U5 | 2.40 | 100.0774 | 6.07889 | 82.0683;100.0776;199.1427 | 2-Hydroxy-2-methylbutanenitrile | C_5_H_10_NO | [M+H]^+^ | Branched amino acid metabolism | ↑** | ↓ |
| U6 | 2.43 | 146.0825 | 4.26048 | 74.0602; 86.0612; 100.0740;128.0734;130.0535; 146.0822 | *N*4-Acetylaminobutanoate | C_6_H_12_NO_3_ | [M+H]^+^ | Arginine and proline metabolism | ↓** | ↑** |
| U7 | 3.52 | 160.0982 | 3.0967 | 114.0946; 142.0891; 160.0983; 319.1876 | Betonicine | C_7_H_14_NO_3_ | [M+H]^+^ | Arginine and proline metabolism | ↓** | ↑* |
| U8 | 3.73 | 160.0981 | 4.65537 | 76.0408;85.0673; 114.0946;142.0891; 160.0983 | Isovalerylglycine | C_7_H_14_NO_3_ | [M+H]^+^ | Branched amino acid metabolism | ↓** | ↑* |
| U9 | 6.24 | 233.0933 | 3.70848 | 233.0934; 465.1868 | 1-Methoxypyrene | C_17_H_13_O | [M+H]^+^ | Polycyclic aromatic hydrocarbon degradation | ↑** | ↓** |
| U10 | 7.35 | 170.0613 | 3.39965 | 142.0641;170.0617;245.0930 | Unkown | C_11_H_7_NO | [M+H]^+^ |  | ↑** | ↓** |
| U11 | 7.54 | 220.1379 | 5.11052 | 97.1026;203.1105; 220.1371 | Pentahomomethionine | C_10_H_21_NO_2_S | [M+H]^+^ | Glucosinolate biosynthesis | ↓** | ↑ |

**Supplementary Table 4-1.** The potential biomarkers of feces in antibiotics-induced gut microbiota dysbiosis rats detected by UPLC-Q-TOF/MS. (*: *p* ＜ 0.05；**：*p* ＜ 0.01)

| **No.** | **RT** | ***m/z*** | **VIP** | **MS/MS** | **Metabolites** | **Formula** | **Adduct ion** | **Pathway** | **M ：C** | **T : M** |
| --- | --- | --- | --- | --- | --- | --- | --- | --- | --- | --- |
| F1 | 1.28 | 131.1185 | 3.03561 | 55.0535; 60.0452; 72.0816; 114.0927; 131.1182 | N-Acetylputrescine | C_6_H_15_N_2_O | [M+H]^+^ | Arginine and proline metabolism | ↑** | ↓ |
| F2 | 1.33 | 204.0874 | 2.7646 | 126.0569;138.0550;144.0664;168.0662;186.0784;204.0879 | N-Acetyl-D-glucosamine | C_8_H_14_NO_5_ | [M+H-H_2_O]^+^ | Amino sugar and nucleotide sugar metabolism | ↓** | ↑ |
| F3 | 1.34 | 137.0470 | 2.79336 | 55.0255;82.0401; 94.0349;110.0351; 119.0330;137.0470 | Hypoxanthine | C_5_H_5_N_4_O | [M+H]^+^ | Purine metabolism | ↓** | ↑** |
| F4 | 1.34 | 222.0977 | 2.6581 | 222.098; 204.0876; 186.0760;162.0749;130.0507 | N-Acetyl-D-mannosamine | C_8_H_16_NO_6_ | [M+H]^+^ | Amino sugar and nucleotide sugar metabolism | ↓** | - |
| F5 | 1.68 | 138.0922 | 6.07088 | 77.0385; 93.0708; 103.0553;121.0656;138.0918 | Tryamine | C_8_H_11_NO | [M+H]^+^ | Tyrosine metabolism | ↑** | ↓ |
| F6 | 1.81 | 173.1292 | 2.96532 | 69.0690;86.0976; 114.0922;132.1029;173.1287 | Isoleucine | C_8_H_17_N_2_O_2_ | [M+ACN+H]^+^ | Branched amino acid metabolism | ↑** | ↓** |
| F7 | 2.19 | 100.0767 | 2.91198 | 55.0577; 70.0674; 72.0796; 100.0769; 118.0856 | Valine | C_5_H_10_NO | [M+H-H_2_O]^+^ | Branched amino acid metabolism | ↑** | ↓ |
| F8 | 3.07 | 205.0976 | 6.39094 | 118.0665;132.0814;144.0819;146.0609;159.0930;170.0617;188.0715;205.0977;409.1873 | Tryptophan | C_11_H_13_N_2_O_2_ | [M+H]^+^ | Tryptophan metabolism | ↑** | ↓ |
| F9 | 3.61 | 208.0612 | 5.45374 | 134.0600;162.0550;190.0500;208.0600 | 2-Formaminobenzoylacetate | C_10_H_9_NO_4_ | [M+H]^+^ | Tryptophan metabolism | ↑** | - |
| F10 | 4.36 | 192.0663 | 2.95135 | 146.0607;192.0657 | 5-Hydroxyindoleacetate | C_10_H_10_NO_3_ | [M+H]^+^ | Tryptophan metabolism | ↑** | - |
| F11 | 5.92 | 595.3492 | 8.91876 | 595.349 | Urobilin | C_33_H_46_N_4_O_6_ | [M+H]^+^ | Porphyrin and chlorophyll metabolism | ↓** | ↑ |

**Supplementary Table 4-2.** The potential biomarkers of feces in antibiotic induced gut microbiota dysbiosis rats detected by UPLC-Q-TOF/MS. (*: *p* ＜0.05；**：*p* ＜ 0.01)

| **No.** | **RT** | ***m/z*** | **VIP** | **MS/MS** | **Metabolites** | **Formula** | **Adduct ion** | **Pathway** | **M : C** | **T : M** |
| --- | --- | --- | --- | --- | --- | --- | --- | --- | --- | --- |
| F12 | 6.91 | 426.3213 | 2.74835 | 363.2890;373.2740; 391.2840;409.2950 | Hyocholate | C_24_H_44_NO_5_ | [M+NH_4_]^+^ | Bile acid metabolism | ↑** | ↓ |
| F13 | 7.06 | 426.3211 | 2.91848 | 363.2890;373.2740; 391.2840;409.2950 | Vulpecholate | C_24_H_44_NO_5_ | [M+NH_4_]^+^ | Bile acid metabolism | ↓** | - |
| F14 | 7.74 | 424.3060 | 8.24187 | 285.1845;371.2590;389.2691;407.2793;424.3069 | 3-Oxocholate | C_24_H_42_NO_5_ | [M+NH_4_]^+^ | Bile acid metabolism | ↑** | ↓* |
| F15 | 8.70 | 426.3214 | 9.67317 | 363.2890;373.2740; 391.2840;409.2950 | Cholate | C_24_H_44_NO_5_ | [M+NH_4_]^+^ | Bile acid metabolism | ↑** | ↓ |
| F16 | 8.71 | 410.3263 | 2.82754 | 355.2632;373.2734;408.3114 | Murideoxycholate | C_24_H_44_NO_4_ | [M+NH_4_]^+^ | Bile acid metabolism | ↓** | - |
| F17 | 9.02 | 785.5918 | 7.03379 | 321.2517;339.2630;357.2777;375.2889;393.2998;785.5885 | Chendeoxycholate | C_48_H_81_O_8_ | [2M+H]^+^ | Bile acid metabolism | ↓** | ↑ |
| F18 | 9.22 | 391.2844 | 2.86207 | 327.2676;355.2647;373.2761;391.2864;408.3129 | Nutriacholate | C_24_H_39_O_4_ | [M+H]^+^ | Bile acid metabolism | ↓** | - |
| F19 | 10.12 | 408.3111 | 8.28248 | 327.2676;355.2647;373.2761;391.2864;408.3129 | 7-Hydroxy-3-oxocholanoate | C_24_H_42_NO_4_ | [M+NH_4_]^+^ | Bile acid metabolism | ↓** | ↑** |
| F20 | 10.12 | 391.2844 | 8.4264 | 327.2676;355.2647;373.2761;391.2864;408.3129 | 7a-Hydroxy-3-oxo-5b-cholanoate | C_24_H_39_O_4_ | [M+H]^+^ | Bile acid metabolism | ↓** | ↑ |
| F21 | 11.39 | 410.3265 | 10.121 | 355.2632;373.2734;408.3114 | Ursodeoxycholate | C_24_H_44_NO_4_ | [M+NH_4_]^+^ | Bile acid metabolism | ↓** | ↑* |
| F22 | 11.39 | 785.5918 | 2.82934 | 321.2517;339.2630;357.2777;375.2889;393.2998;785.5885 | Deoxycholate | C_48_H_81_O_8_ | [2M+H]^+^ | Bile acid metabolism | ↓** | ↑ |
| F23 | 11.56 | 408.3110 | 8.02338 | 355.2632;373.2734;408.3114 | 12-Ketodeoxycholate | C_24_H_44_NO_4_ | [M+NH_4_]^+^ | Bile acid metabolism | ↓** | - |
